# A systematic evaluation of deep learning methods for the prediction of drug synergy in cancer

**DOI:** 10.1371/journal.pcbi.1010200

**Published:** 2023-03-23

**Authors:** Delora Baptista, Pedro G. Ferreira, Miguel Rocha

**Affiliations:** 1 CEB - Centre of Biological Engineering, University of Minho, Braga, Portugal; 2 LABBELS - Associate Laboratory, Braga, Guimarães, Portugal; 3 Department of Computer Science, Faculty of Sciences, University of Porto, Porto, Portugal; 4 INESC TEC, Porto, Portugal; 5 Ipatimup - Institute of Molecular Pathology and Immunology of the University of Porto, Porto, Portugal; 6 i3s - Instituto de Investigação e Inovação em Saúde da Universidade do Porto, Porto, Portugal; Academy of Mathematics and Systems Science, Chinese Academy of Science, CHINA

## Abstract

One of the main obstacles to the successful treatment of cancer is the phenomenon of drug resistance. A common strategy to overcome resistance is the use of combination therapies. However, the space of possibilities is huge and efficient search strategies are required. Machine Learning (ML) can be a useful tool for the discovery of novel, clinically relevant anti-cancer drug combinations. In particular, deep learning (DL) has become a popular choice for modeling drug combination effects. Here, we set out to examine the impact of different methodological choices on the performance of multimodal DL-based drug synergy prediction methods, including the use of different input data types, preprocessing steps and model architectures. Focusing on the NCI ALMANAC dataset, we found that feature selection based on prior biological knowledge has a positive impact—limiting gene expression data to cancer or drug response-specific genes improved performance. Drug features appeared to be more predictive of drug response, with a 41% increase in coefficient of determination (R^2^) and 26% increase in Spearman correlation relative to a baseline model that used only cell line and drug identifiers. Molecular fingerprint-based drug representations performed slightly better than learned representations—ECFP4 fingerprints increased R^2^ by 5.3% and Spearman correlation by 2.8% w.r.t the best learned representations. In general, fully connected feature-encoding subnetworks outperformed other architectures. DL outperformed other ML methods by more than 35% (R^2^) and 14% (Spearman). Additionally, an ensemble combining the top DL and ML models improved performance by about 6.5% (R^2^) and 4% (Spearman). Using a state-of-the-art interpretability method, we showed that DL models can learn to associate drug and cell line features with drug response in a biologically meaningful way. The strategies explored in this study will help to improve the development of computational methods for the rational design of effective drug combinations for cancer therapy.

## Author summary

Cancer therapies often fail because tumor cells become resistant to treatment. One way to overcome resistance is by treating patients with a combination of two or more drugs. Some combinations may be more effective than when considering individual drug effects, a phenomenon called drug synergy. Computational drug synergy prediction methods can help to identify new, clinically relevant drug combinations. In this study, we developed several deep learning models for drug synergy prediction. We examined the effect of using different types of deep learning architectures, and different ways of representing drugs and cancer cell lines. We explored the use of biological prior knowledge to select relevant cell line features, and also tested data-driven feature reduction methods. We tested both precomputed drug features and deep learning methods that can directly learn features from raw representations of molecules. We also evaluated whether including genomic features, in addition to gene expression data, improves the predictive performance of the models. Through these experiments, we were able to identify strategies that will help guide the development of new deep learning models for drug synergy prediction in the future.

## Introduction

The phenomenon of drug resistance is one of the greatest challenges in the fight against cancer. Although many tumors initially respond well to a given treatment, the efficacy of single-drug anti-cancer therapies is often diminished due to the existence of tumor drug resistance mechanisms. Resistance-conferring characteristics may already be present in the tumor cells prior to therapy, or they may arise as an adaptive response of the tumor to the treatment itself [1]. One of the main drivers of resistance is intratumoral heterogeneity. Genomic instability in cancer leads to the emergence of subpopulations of cells within a tumor with distinct characteristics and different sensitivity to drugs. Treatment may exert selective pressure on the cells and select subpopulations possessing characteristics that favor drug resistance, leading to future relapse [2].

Combining multiple treatments instead of administering a single drug can help to reduce drug resistance [3]. Drug combinations may circumvent pre-existing resistance mechanisms more easily and prevent the development of acquired resistance mechanisms [4]. In addition, certain combinations may be more effective than would be expected when taking into account the effects of each of the constituent compounds on their own, a phenomenon called drug synergy. Drug synergy can increase treatment efficacy without requiring an increase in drug dosage, potentially avoiding an increase in toxicity as well [5]. Synergistic interactions can arise through a variety of mechanisms: the compounds in a combination may have the same target, different targets belonging to the same pathway/biological process, or different targets belonging to related pathways (S1 Fig) [6]. Drug combinations may also produce effects that are greater than expected when the activity of one of the drugs enhances the transport, permeation, distribution or metabolism of the other drug [6]. Synergy is quantified based on the difference between the observed effect of a given drug combination and the expected combination effect determined using a reference model [7]. Combination effects are then usually summarized as a single synergy score per drug pair, taking all of the tested doses into consideration. Classifying combinations as synergistic based on synergy scores is not straightforward, however. Multiple drug combination reference models exist, each with different underlying assumptions, which can lead to contradictory results [7, 8]—a drug combination that would be considered synergistic according to one reference model might be classified as antagonistic according to another. A given reference model may lead to erroneous conclusions when the mechanism behind the drug interaction fails to adhere to its assumptions [7]. In addition, if synergy is considered a feature of each dose pair combination instead of a characteristic of the drug pair itself, different conclusions regarding synergy may be reached [8]. Differences in experimental design [7, 8] and the dose-response profiles of individual compounds [7] may also affect the determination of combination effects.

Novel effective anti-cancer drug combinations can be discovered using high-throughput cell viability assays. In these assays, a large number of candidate drug combinations are screened at different concentrations across a panel of cancer cell lines and the cellular response to the drug is measured. In recent years, several datasets from large-scale drug screening initiatives have been made publicly available [9–11]. The largest of these is the National Cancer Institute (NCI) A Large Matrix of Anti-Neoplastic Agent Combinations (ALMANAC) project [10], which screened over 5,000 pairs of FDA-approved drugs against National Cancer Institute 60 Human Cancer Cell Line Screen (NCI-60), a panel of 60 tumor cell lines that have been extensively characterized at the molecular level [12]. The project uncovered several synergistic drug pairs, including two clinically novel combinations that are currently being evaluated in phase I clinical trials [10].

Despite the existence of these high-throughput technologies, screening all conceivable drug combinations is still infeasible, for both practical and financial reasons [11, 13]. Computational methods could greatly reduce the search space, thus minimizing the experimental effort required to find truly effective anti-cancer drug combinations. Biological network analysis-based approaches can be used to prioritize drug combinations and study the underlying mechanisms of joint action [14–16]. Another alternative is to use ML methods to model the response of cells to drug combinations. ML can be used to learn functional mappings between very high-dimensional input data and a score that reflects drug combination effects. This makes it a powerful approach to develop models that are able to predict drug synergy based on drug combination screening experiments and other relevant data. Several ML models for drug synergy prediction have been described in the literature [11, 17–21]. Many of these studies used tree-based ML methods, such as random forests (RFs) [17, 18, 20] or gradient boosting [18, 19, 21]. ML approaches for drug synergy prediction are usually developed using large-scale, publicly available drug combination screening data and omics datasets characterizing the screened cancer cell lines. Some of these resources are listed in Table 1, and we refer readers to other articles [22, 23] for information on additional resources.

**Table 1:** Publicly available high-throughput drug combination screening datasets and large-scale cancer cell line genomics and transcriptomics datasets that can be used to develop drug synergy prediction models. The datasets that were used in this work are highlighted in bold.

Data Type	Dataset	Website	Cell Lines	Drugs	Pairwise Combinations	Drug Pair + Cell Line Triplets	Features
**Drug combination screen**	**NCI ALMANAC [10] (CellMinerCDB) [24]**	https://discover.nci.nih.gov/rsconnect/cellminercdb/	**60**	**104**	**5,355**	**306,365**	-
Drug combination screen	Merck Compound Screen [9]		39	38	583	22,737	-
Drug combination screen	AstraZeneca [11]		85	118	910	11,576	-
**RNA-Seq (FPKM)**	**NCI-60 [25] (CellminerCDB [24])**	https://discover.nci.nih.gov/rsconnect/cellminercdb/	**60**	-	-	-	**23,826**
RNA-Seq (TPM)	CCLE [26] (DepMap)	https://depmap.org/portal/download/	1,406	-	-	-	53,970
RNA-Seq (FPKM)	Cell Model Passports [27]	https://cellmodelpassports.sanger.ac.uk/downloads	1,293	-	-	-	37,602
RNA-Seq (TPM)	Cell Model Passports [27]	https://cellmodelpassports.sanger.ac.uk/downloads	1,293	-	-	-	37,602
**Mutations**	**NCI-60 [28] (cBioPortal [29, 30])**	https://www.cbioportal.org/study/summary?id=cellline_nci60	**67**	-	-	-	**13,393**
Mutations	CCLE [26] (DepMap)	https://depmap.org/portal/download/	1,771	-	-	-	18,784
Mutations	Cell Model Passports [27]	https://cellmodelpassports.sanger.ac.uk/downloads	1,283	-	-	-	20,472
**Copy number (GISTIC)**	**NCI-60 [28] (cBioPortal [29, 30])**	https://www.cbioportal.org/study/summary?id=cellline_nci60	**60**	-	-	-	**19,062**
Copy number (GISTIC)	Cell Model Passports [27]	https://cellmodelpassports.sanger.ac.uk/downloads	978	-	-	-	20,669

One particular subset of ML that has attracted great interest from researchers in this field is deep learning (DL). These are models composed of multiple processing layers [31], giving them the ability to learn complex, non-linear functions. Furthermore, unlike most traditional ML methods, DL approaches typically do not require extensive feature selection before training, since they have the ability to learn higher-order representations directly from raw input data [32]. Since DL models can handle large amounts of high-dimensional and noisy data, they are good candidates for the development of drug synergy prediction models.

Preuer et al. [33] presented DeepSynergy, a feedforward, fully-connected deep neural network that uses chemical features and gene expression data to predict drug synergy. Xia et al. developed a multimodal DL model to predict the growth inhibition of cell lines from the NCI ALMANAC project [34]. This model includes separate feature-encoding subnetworks for each input data type (drug descriptors, gene expression, microRNA and proteomics data) and a cell line growth prediction network. Several other DL-based drug synergy prediction models have since been reported in the literature. Similar to the model proposed in 2018 by Xia et al., many of these more recent models adopt a multimodal architecture [35–38].

Beyond fully connected models [33, 34, 36, 37, 39], other innovative architectures have been proposed. Zhang et al. [40] developed a sparsely-connected deep belief network constrained by biological prior knowledge. The recent architecture of the TranSynergy model [41] includes a transformer [42] component, as well as fully connected layers. A method called REpresentation of Features as Images with NEighborhood Dependencies (REFINED) was developed to transform drug descriptors into images, so that convolutional neural networks (CNNs) could be used to model drug synergy instead of the typical fully connected networks [43]. Another study used graph neural networks (GNNs) for drug-specific subnetworks to learn drug representations directly from the compound structures in an end-to-end manner [38]. Several recent studies have used GNNs trained on graphs containing information on interactions between the drugs in a combination, between drugs and their targets, and/or interactions between genes or proteins in the cell lines [44–47].

Most drug synergy prediction models use drug features or gene expression features or a combination of both. Other models include additional cell line information, such as genetic data (somatic mutations and/or copy number variations (CNVs)) [35, 40] or proteomics data [34, 39]. Drug target-specific features have also been included [37, 40, 41]. Since adding more features increases the complexity of the models, assessing which types of input data are more informative and predictive of drug synergy is essential.

Precomputed molecular descriptors or fingerprints are used as chemical features to represent the drugs, as an alternative to the use of end-to-end DL methods to learn the relevant compound features directly from the compound structures. Given that the screening datasets that are currently available only contain a very limited number of compounds, it is still unclear whether there is any benefit in using learned representations instead of traditional fingerprints and descriptors. A recent study benchmarked several compound representations on a large drug synergy dataset and found that DL-based representations were able to outperform traditional fingerprints [21]. However, the authors also noted that the difference between the top performing DL-based methods and the best fingerprints was not substantial and that other concerns, such as interpretability, may be more important.

Feature reduction is often applied to the cell line omics data, either by using specific gene lists [38, 40, 41], or by employing unsupervised data dimensionality reduction techniques, such as principal component analysis (PCA) [39, 48] or autoencoders [35, 39]. Using predefined gene lists to select features provides greater control over the selection process and might make the models easier to interpret biologically. However, certain approaches, such as limiting the gene features to known drug targets present in the training set, may limit the generalization of the models. Data-driven approaches avoid this problem.

Another advantage of data-driven dimensionality reduction techniques is the capacity to be trained using much larger datasets with data from more cell lines than those used in the screening datasets [39], or even patient data [35]. Nevertheless, a limitation of this approach is the difficulty in interpreting the results. Therefore, evaluating which feature reduction methods are capable of achieving satisfactory performance, as well as simultaneously facilitating model interpretability, is an essential step when designing drug synergy prediction models.

The impact of different methodological aspects on the drug synergy prediction models is still unclear and a systematic evaluation is missing. In this work, we set out to investigate the impact of different methodological variables on the performance of drug synergy prediction DL methods, using the ALMANAC drug combination screening dataset. Namely, we evaluated the impact of different preprocessing steps, types of input data, and DL architectures on the final performance of the methods. Prior biological knowledge was used to select cell line features and to facilitate model interpretation.

Interpretability is an important requirement of biomedical predictive systems. We further explored recent methodologies to determine the importance of features and the interpretability of the prediction mechanisms.

We were able to identify the types of input data that are more predictive of drug response, as well as the feature selection and data representation methods that produce the best results. We also found that combining different models improves performance. Additionally, we demonstrate that the decisions made by the DL models are driven by biologically meaningful features.

The remainder of this article is structured as follows: the Results section briefly summarizes the different models and methodological choices that were tested in this work, and reports the results of these tests. It also includes a subsection focusing on model interpretability. In the Discussion section, the main findings of this study, as well as its limitations, are discussed. The research methodology is described in detail in the Materials and Methods section at the end of the article.

## Results

### Testing the impact of different methodological variables

**Fig 1:**
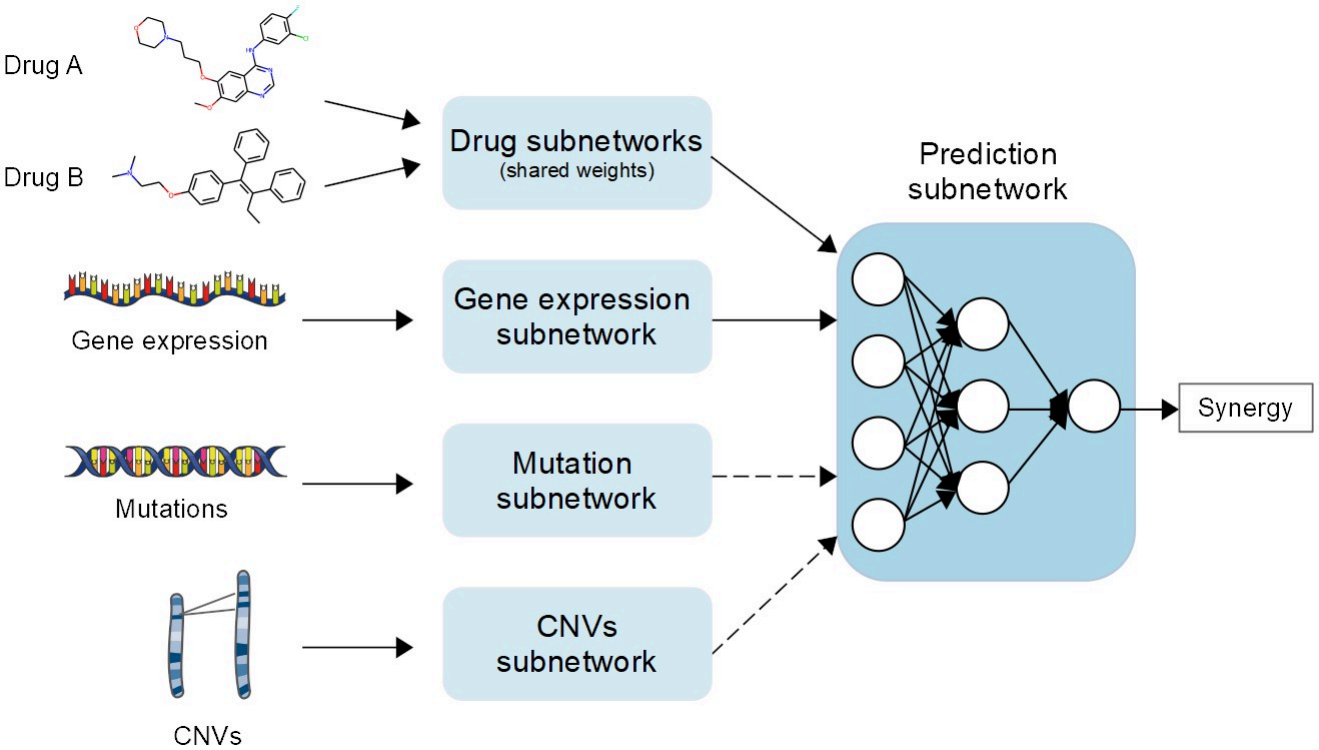
A representation of the general architecture of the multimodal DL models developed in this study. A model is defined as a combination of the learning algorithm itself and the data preparation steps required beforehand.

We developed several multimodal DL models (Fig 1) to predict drug combination effects summarized as ComboScores, using the ALMANAC dataset [10]. In total, 24 different DL models and 6 ML models were developed. A detailed description of the models is provided in the Materials and Methods section, and S2 Fig provides a summary of the different methodological choices that were tested. The results of these tests will be described in the following subsections.

### Baseline models

The baseline models that were built served as references for subsequent models. All of the models developed in this study performed better than a random baseline model, that always predicts the average ComboScore value of the training set (S1 Table).

**Fig 2:**
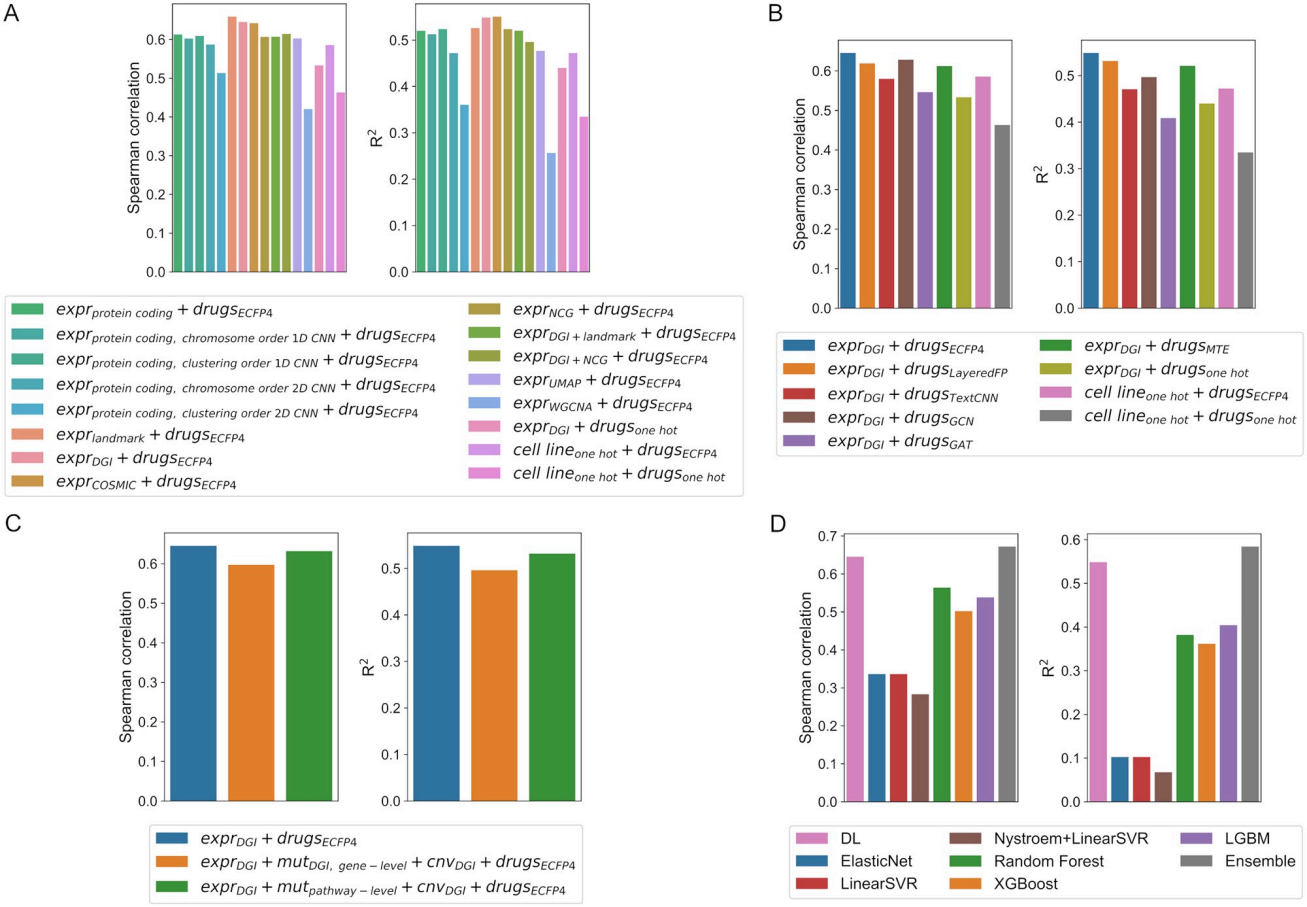
Performance scores (Spearman correlation—left—and *R*^2^—right) of the different models tested in this study. (A) Performance scores of models with different gene expression feature-encoding subnetworks. (B) Performance scores of models with different drug encoding subnetworks. (C) Performance scores of models trained with and without mutation (*mut*) and copy number variation (*cnv*) data in addition to gene expression and drug features. (D) Performance scores of non-DL models compared with one of the best DL models developed in this study.

To assess the importance of including a certain input data type, we analyzed the performance of models trained on one-hot encoded identifiers instead of actual cell line or drug features:

- *cell line_one hot_ + drugs_one hot_*—a model trained exclusively with one-hot encoded cell line and drug identifiers, which was used to determine if omics and chemical features include information that is relevant for the prediction of drug synergy, beyond the information contained in drug or cell line identifiers;
- *cell line_one hot_ + drugs_ECFP4_*—a model using one-hot encoded cell line identifiers and extended connectivity fingerprint (ECFP)4 fingerprints as input features, to determine the impact of removing cell line omics features on the performance of the model;
- *expr_DGI_ + drugs_one hot_*—a model using one-hot encoded drug identifiers and genes with known or potential interactions with drugs screened in the ALMANAC project (drug-gene interaction (DGI) genes) as features, to determine the impact of removing drug features;

Nearly all of the models performed better than the *cell line_one hot_ + drugs_one hot_* model (Fig 2). Using one-hot encoded cell line names instead of omics features (*cell line_one hot_ + drugs_ECFP4_* model) decreased model performance (Fig 2A). Nevertheless, the scores are comparable to those of some of the models that had been trained on actual gene expression data. When using one-hot encoded drug identifiers instead of chemical features as input (*expr_DGI_ + drugs_one hot_* model), model performance dropped even more (Fig 2B). Both the (*cell line_one hot_ + drugs_ECFP4_* model and the *expr_DGI_ + drugs_one hot_* model performed better than the *cell line_one hot_ + drugs_one hot_* model. These results seem to indicate that both drug and gene expression features contribute to the predictive capacity of the model, and that drug features appear to be more predictive of drug synergy than omics features.

### Different gene expression subnetworks

For the most part, models with fully connected gene expression subnetworks trained using the original gene expression values (tabular format) outperformed 1D and 2D convolutional gene expression subnetworks (Fig 2A). The 1D CNN subnetworks were able to achieve performance scores that were similar to some of the lower ranked fully connected gene expression subnetworks, but the 2D CNN subnetworks performed worse. This was independent of the order of genes obtained by clustering or by chromosome position. In light of these results, we only used fully connected layers for the omics subnetworks in subsequent tests.

The best gene expression subnetworks were fully connected subnetworks trained on the log-transformed and min-max scaled Fragments Per Kilobase of transcript per Million mapped reads (FPKM) values, with genes being selected according to predefined gene lists (Fig 2A). The top three models were trained on expression data limited to landmark genes, Catalogue of Somatic Mutations in Cancer (COSMIC) cancer genes or DGI genes. Working with the full set of over 18,000 protein coding genes present in this dataset was not particularly advantageous. Most of the feature selection methods that used smaller gene lists produced models with similar or higher performance scores.

Using dimensionality reduction led to worse performance (Fig 2A). Uniform Manifold Approximation and Projection (UMAP) embeddings were able to perform similarly to some of the gene list selection methods, while using Weighted Gene Co-expression Network Analysis (WGCNA) as a dimensionality reduction method resulted in poorer performance, worse than the baseline models trained on one-hot encoded cell line names.

In the following sections, we will only focus on models using *expr_DGI_* as the feature-encoding subnetwork for gene expression data. This gene selection method ranked second in terms of scoring metrics (R^2^ and Spearman correlation), and might also provide better interpretability.

### Drug encoding networks

The model trained on ECFP4 fingerprints outperformed all of the other subnetworks in terms of all scoring metrics (Fig 2B). Another fingerprint-based drug encoding scheme, RDKit layered fingerprints (LayeredFP), achieved the second-best R^2^ score, while the graph convolutional network (GCN) subnetwork achieved the second-best Spearman correlation score. In general, most of the drug subnetworks achieved similar performance scores, minus a few exceptions (such as the graph attention network (GAT) subnetwork).

### Additional cell line features

Including mutation and CNV data slightly decreased model performance (Fig 2C). The model trained on pathway-level mutation features performed slightly better than the model trained using gene-level mutation data. This may be due to the fact that more genes were taken into consideration when summarizing the mutation data at the pathway level, which would be an indication that relevant genetic information is being lost when only DGI genes are considered. The mutation dataset summarized at the pathway-level is also less sparse (i.e. a lower percentage of entries are zero) than the gene-level dataset.

### Comparison with other machine learning models

To determine if there is any advantage in using DL instead of other ML algorithms to model drug combination effects, we compared our DL model trained on gene expression features of DGI genes and ECFP4 fingerprints (*expr_DGI_ + drugs_ECFP4_* model) against 5 different ML models trained on the same features (Fig 2D). We tested elastic net, linear support vector regression (SVR) and linear SVR preceded by a radial basis function (RBF) kernel approximation method, RF, extreme gradient boosting (XGBoost) light gradient boosting machine (LGBM) models. The DL model outperformed all of the ML models. The best non-DL models were LGBM (in terms of R^2^) and RF (in terms of Spearman correlation). The performance of all of the tree-based models (RF, XGBoost and LGBM) is on par with some of the lower ranking DL models described in previous sections. The elastic net and SVR models did not perform well, with worse performance than the baseline DL model trained on one-hot encoded identifiers.

### Heterogeneous ensemble

We also created a simple heterogeneous ensemble, to determine if combining different DL approaches, as well as other ML models, could lead to better results. The ensemble outperformed the best individual DL models for both scoring metrics (R^2^ = 0.584 and Spearman correlation = 0.672 vs. R^2^ = 0.549 and Spearman correlation = 0.645 for the *expr_DGI_ + drugs_ECFP4_* model, for example). These results show that combining different DL architectures and different learning algorithms can improve the generalizability of drug synergy prediction models.

### Feature importance

One of the main disadvantages of using DL models to predict drug response is that they are difficult to interpret. The SHapley Additive exPlanations (SHAP) [49] interpretability framework was used to determine which features were the most important for the *expr_DGI_ + drugs_ECFP4_* model. SHAP values reflect the contribution of each feature to a prediction.

**Fig 3:**
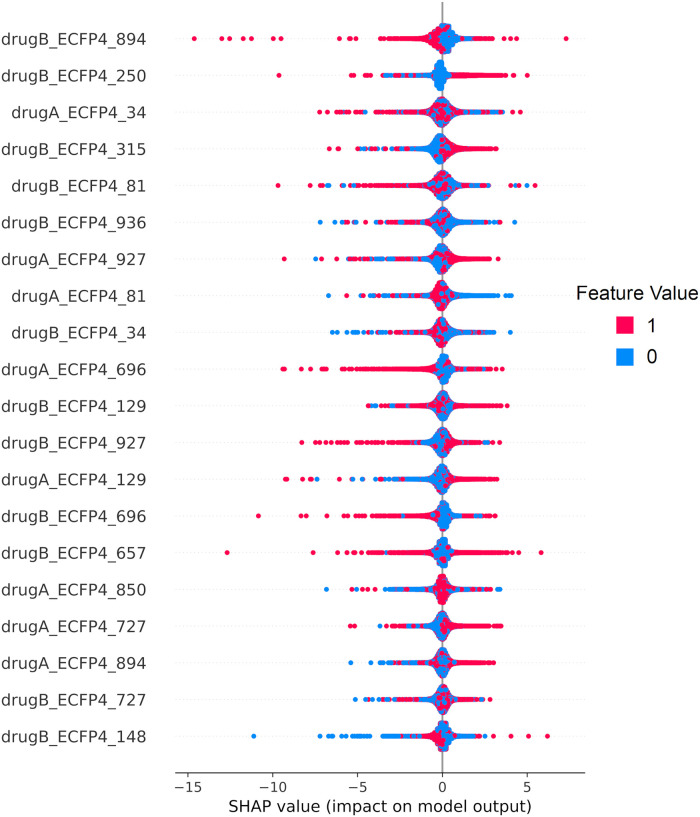
Top 20 most important features, ranked by mean absolute SHAP values.

Fig 3 shows the top 20 most important features. Important features are those that have greater absolute SHAP values. Each point in the figure represents an instance in the test set. The points are distributed along the X-axis according to the SHAP values. The colors represent the feature values. This reveals how the value of a feature influences the model output for a given sample. For example, when ECFP4 bit 250 is “ON” (value = 1), the impact on the model output is usually positive, pushing the predicted ComboScore higher.

All of the top 20 features for this DL model are drug features. A similar result is obtained for the RF model (S2 Table). In addition, when grouping features by input type, the corresponding mean absolute SHAP values of each of the groups also suggest that drug features are the most important type of features (Table 2). These results seem to indicate that, for the ALMANAC dataset, drug features are the most relevant for drug response prediction.

**Table 2:** Mean absolute SHAP values for features grouped by input type. SHAP values for groups of features were calculated by summing the SHAP values of all of the individual features, and then the mean absolute SHAP value across all samples was calculated for the grouped features.

Input type	Mean absolute SHAP value for grouped features
expr	10.64
drugA	14.79
drugB	17.18

The atom environments that define each of the ECFP4 bits that appear among the top 20 most important features are shown in S3 Fig. The atoms that are at the center of the atom environment are highlighted in blue. Atoms and bonds that appear in light gray are parts of the molecule that are not part of the fingerprint bit, but influence the central atom’s connectivity invariants. Several of the top bits correspond to simple atom environments (environments with a radius of 0, which only include the central atom itself, or a radius of 1), which will be set to 1 for many of the drugs in the dataset. Many bits have more than one associated structure. Some of these are similar atom environments with slight differences in terms of the adjacent parts of the molecules that are not part of the fingerprint. Other cases are bit collisions, indicating that there is some loss of information when using 1024-bit fingerprints.

**Fig 4:**
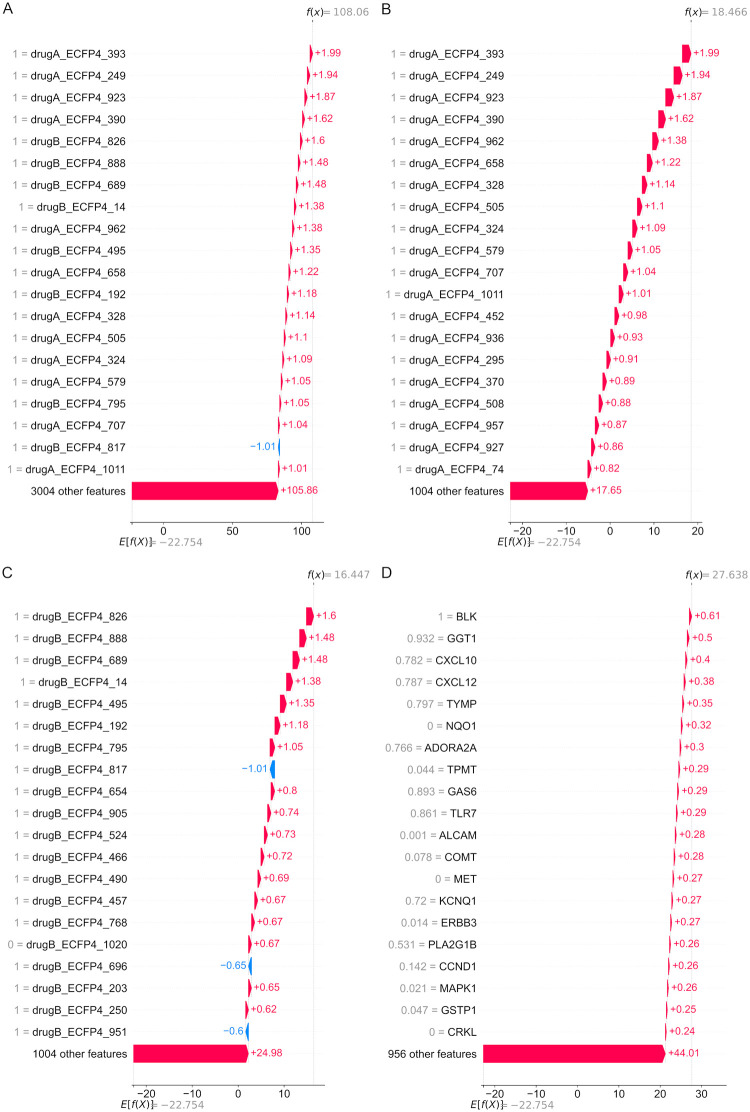
Most important features (ranked by absolute SHAP values) for test set example 11835, shown as waterfall plots. The plot starts at a base value of -22.754, which is the expected model output (determined based on a background dataset). Each row shows how each feature positively or negatively contributes to move the value from the expected output to the predicted value for this sample. (A) Top 20 features (from all features). (B) Top 20 drugA features. (C) Top 20 drugB features. (D) Top 20 gene expression features.

SHAP can also be used to provide explanations for specific examples. We analyzed a specific example (11835) that involved the SR cell line, derived from an anaplastic large cell lymphoma [26]. The drugs screened in this experiment were Topotecan hydrochloride, which interacts with the topoisomerase I-DNA complex at the site where the DNA cleavage occurs [50], and Gefitinib, which inhibits the epidermal growth factor receptor (EGFR) tyrosine kinase domain [51]. Gefitinib may enhance the therapeutic activity of Topotecan by affecting the transport of the drug out of the cells [52]. This experiment was selected for further analysis since this combination was found to have greater-than-additive effects in the SR cell line in the ALMANAC screen (ComboScore = 108.00), and because the ComboScore predicted by our model (ComboScore = 108.06) was close to the true value. Fig 4A displays the explanation of the prediction for this case. Each row shows the contribution of each feature to improve the prediction score. For this specific example, all of the top 20 features were, once again, ECFP4 fingerprint bits from drugA and drugB.

The SHAP values for each of the different input types were also analyzed separately. Fig 4B and 4C show the 20 most important ECFP4 bits for drugA (Topotecan hydrochloride) and drugB (Gefitinib), respectively, along with the corresponding SHAP values. The 2D structures of 15 of the most important “ON” bits (value = 1) that contributed positively are shown in S4 Fig for Topotecan and S5 Fig for Gefitinib.

As shown in S4 Fig, bits 249 and 658 capture parts of the lactone ring in Topotecan which establishes an important interaction with Topoisomerase I via hydrogen bonding [50]. Many of the remaining bits capture parts of the planar ring system that allows the drug to intercalate between two DNA base pairs and establish pi stacking interactions with the neighboring DNA bases [50]. These results reveal that our model was able to correctly identify which substructures are most important for the bioactivity of Topotecan.

The DL model was also able to identify features in Gefitinib that are more predictive of drug response and are consistent with the literature (S5 Fig). Bits 795, 490 and 203 capture parts of the quinazoline core that are involved in an important drug-protein interaction, with bit 203 focusing on the nitrogen atom that interacts with the adenosine triphosphate (ATP) binding site of EGFR via a hydrogen bond [53]. The majority of the bits in S5 Fig, however, correspond to the morpholine substituent or the 3-chloro-4-fluoro aniline substituent, which are not directly involved in drug-protein interactions [53]. The DL model may have identified these features as more important because they might be structures that distinguish Gefitinib from other compounds in the ALMANAC dataset, instead of being important for the drug activity *per se*.

The top 20 gene expression features are shown in Fig 4D. None of the genes that encode the primary targets of Topotecan and Gefitinib are found among the top 20 gene expression features. *TOP1*, which encodes DNA Topoisomerase I, was not present in the list of drug-gene interactions for Topotecan hydrochloride in The Drug-gene Interaction Database (DGIdb). The gene encoding DNA Topoisomerase I Mitochondrial (*TOP1MT*) was present in the list of drug-gene interactions, but it was only the 246th most important gene expression feature. EGFR ranked higher, being the 46th most important gene expression feature in this experiment. This may suggest that the synergistic interaction between Topotecan and Gefitinib in the SR cell line might be the result of off-target interactions instead of interactions of the drugs with their primary targets, although the information provided by the feature importance analysis is not enough to reach a definitive conclusion. Furthermore, none of the genes involved in the drug-gene interactions with Topotecan hydrochloride listed in DGIdb are among the top 20 most important gene expression features. Four of the top 20 genes (*BLK*, *MET*, *ERBB3* and *CRKL*) are involved in drug-gene interactions with Gefitinib, however. Gefitinib has been experimentally shown to be capable of binding to *BLK* [54], although with a much lower affinity than for EGFR. Since the expression of *BLK* is high in the SR cell line, the high SHAP value of *BLK* might be an indication that this interaction may be important for the response of the cells to Gefitinib. Amplification of *MET* [55] or *CRKL* [56] has been linked to acquired resistance to EGFR inhibitors, and *ERBB3* has also been implicated in resistance to EGFR tyrosine kinase inhibitors [55]. Since these genes are less expressed in the SR cell line, the higher SHAP values may be an indication that lower expression of these genes is essential for the cell line to be responsive to treatment with Gefitinib.

Interestingly, several of the most important genes have some kind of connection to the immune system. *CXCL10* and *CXCL12* are both chemokines involved in the recruitment of lymphocytes [57, 58]. *ADORA2A* plays a role in the regulation of the immune system [59], *TLR7* is involved in immune response [60], and *ALCAM* also plays a role in immunity [61]. It is possible that the DL model identified these features as being essential for the prediction of drug synergy because the expression of these genes uniquely identifies the cell line in some way (since SR is a lymphoma cell line, derived from lymphocytes), and not necessarily due to the relevance of these genes for drug response.

A pre-ranked gene set enrichment analysis (GSEA) [62] was performed for test set example 11835 to determine if there is an enrichment of specific gene ontology (GO) terms among the most important genes. Genes were ranked based on their SHAP values. In total, the analysis identified 28 enriched gene sets, most of which refer to cell motility, cell death, and the regulation of kinase or transferase activity (S3 Table). We found that the gene sets with the highest normalized enrichment score (NES) values are leukocyte/lymphocyte-specific GO terms (S3 Table). We also observed that several of the enriched gene sets include the *EGFR* gene, which encodes the protein targeted by Gefitinib (S3 Table).

## Discussion

The results of this study suggest that drug features are more predictive of drug combination effects than cell line features, at least for the ALMANAC dataset, in line with previous results [34]. Substituting cell line identifiers for actual gene expression data (*cell line_one hot_ + drugs_ECFP4_*) produced a model with performance scores that were similar to those of models trained on actual gene expression data. This may be an indication that the expression features are mainly being used by the models as a way to distinguish between cell lines, just as the cell line identifiers. The ability to identify cell lines based on their gene expression profiles is already a positive result, but it does not seem that the decisions made by the DL models are being driven by the identification of specific synergy biomarkers. In addition, the inclusion of other cell line features besides gene expression data was not beneficial. Similar results were obtained in other drug response prediction studies [63, 64].

Several of the compound representation methods tested in this work produced models with similar performance. This finding is in agreement with a recent study by our group that compared the performance of different compound representations across different drug response prediction tasks and concluded that most representations perform similarly [65]. Since different drug representations may capture different characteristics of the compounds that may be equally predictive of drug response, the combined use of different types of drug representations might lead to an improvement in model performance and could be an interesting strategy to explore in the future.

Using prior biological knowledge for feature selection proved to be beneficial. Besides the cancer-specific and DGI gene lists that were utilized in this study, other cancer-specific gene lists and combinations of lists might provide even better results. Pathway propagation methods have been employed in other drug response prediction studies to simulate response to treatment [66] and to extend the selection of genes beyond known drug targets [41]. This strategy was not explored in the current study, but it might also be a way to improve the predictive capacity of the models. It is important to note that using drug targets or DGIs to select genes may limit the generalizability of the model, as new compounds may have DGIs that are different from the ones that were considered relevant for the training set.

Another approach that was not explored in this study is directly including biological knowledge in the neural network by representing the experiments as heterogeneous graphs and modeling drug response using GNNs. Since the number of unique compounds and cell lines in the ALMANAC dataset is relatively small, it is unclear if the models would benefit from the added complexity of this approach.

Despite training the models on a relatively large screening dataset, comprising over 200,000 screening experiments, there are only 59 cell lines and 104 drugs in ALMANAC. The number of unique compounds screened in the ALMANAC project is relatively small, a fact that might explain why fingerprint-based models performed slightly better than learned representations in this case, as learned representations are known to struggle when faced with smaller compound sets [67]. Considering the small number of unique cell lines and drugs, pre-training the feature encoding subnetworks using larger sets of compounds and larger omics datasets obtained from other sources could help improve model performance. Training models on larger and more diverse drug combination datasets from databases such as DrugCombDB [68] or DrugComb [69], which integrate data from several high-throughput screening experiments, could be another strategy to obtain models with better generalization capacity. However, it is important to note that different screening protocols might make it difficult to compare the synergy scores calculated for different screening datasets and to use them jointly to train models. Furthermore, integrating omics datasets from different studies requires the selection of adequate batch correction methods, which might not be straightforward [70].

Our models (as most ML-based synergy prediction models) were trained using data from a cell line screen. This may limit the clinical applicability of these models, as conclusions drawn from *in silico* studies based on cell line screens often fail to translate to the clinic. Cell lines are not always representative of their primary cancer types [71–73], due to mislabeling and contamination, genetic drift, or changes arising from cell culture, among other factors [74]. In addition, the use of cell line data ignores the influence of the tumor microenvironment on drug response [74], as well as the possible adverse systemic effects of drugs. Nevertheless, cell line screens are currently the only option providing sufficient data for the development of DL-based drug synergy prediction methods. The use of transfer learning techniques could help lessen the gap between cell line screens and the clinic, however.

In this study, we focused solely on the prediction of drug synergy. However, assessing the sensitivity of drug combinations is also essential when prioritizing combination therapies [75]. Some combinations may be clinically effective without being synergistic [76], and it has also been noted that there may be a trade-off between synergy and efficacy [77]. In the future, the DL models developed in this work could easily be extended to simultaneously predict a synergy score and a drug combination sensitivity score.

We found that creating simple heterogeneous ensembles comprising both DL and ML models can improve performance. Preto et al. had previously observed that heterogeneous ensembles produced better classifiers than individual models when using the ALMANAC dataset [39], and other synergy prediction studies using different datasets also found that combining multiple models is beneficial [11, 13].

Although SHAP has allowed us to gain more insight into the model and to try to interpret its predictions in a biologically meaningful way, it is still difficult to explain how all of the most important features interact in a particular screening experiment and give rise to drug synergy. Interpretability methods allow us to explain how a DL model works, but they do not necessarily explain the underlying biology/chemistry. It is, therefore, unlikely that the methodology used in our study will be able to uncover the mechanisms underlying drug synergy for particular <*cell line* − *drugA* − *drugB*> triplets, but our findings do confirm that DL models are able to learn to associate important structural features of the compounds and tissue-specific gene expression patterns with drug response. Alternative interpretability methods that are better able to explain higher order interactions between features should be explored in future drug synergy prediction studies.

One important aspect to be considered is the validation scheme that is chosen. In our study, the data were randomly split into training, validation and test sets. Using a different validation scheme, such as a leave-drug-combinations-out approach (i.e. drug combinations that appear in one set are not included in the other sets), or even a leave-drugs-out or leave-cell-lines out validation scheme would likely result in lower performance scores. In addition, the dataset was only split into training, validation and test sets once. Instead of evaluating the models on a single test set, further research should use cross-validation or other resampling methods to estimate uncertainty and to allow statistical comparisons to be made between models. Finally, the models were validated with data from within the same drug combination screening study. Although this is a common model evaluation strategy, cross-validation within a single study has been shown to overestimate model generalizability [78]. In the future it would be interesting to assess different DL modeling strategies by performing a cross-study evaluation of the models as well [78].

## Conclusion

In this study, we performed a systematic analysis of the impact of different methodological choices on the predictive performance of DL-based drug synergy prediction models, to determine which preprocessing and modeling approaches provide the best results. Different input data type combinations, drug encoding schemes, gene expression feature selection/reduction methods and DL architectures were tested, and an ensemble combining the top methods was also evaluated. These experiments enabled the identification of several strategies that may be interesting starting points for the development of new DL-based drug synergy prediction models in the future.

## Materials and methods

### Datasets and data preprocessing

ALMANAC drug response data in the form of ComboScores for <*cell line*, *drugA*, *drugB*> triplets were downloaded from CellMiner Cross Database (CellMinerCDB) [24] (version 1.2). The ComboScore for a given <*cell line* − *drugA* − *drugB*> triplet is the sum of the differences between the expected and observed cell line growth calculated for each dose combination tested in the screen, with expected effects being determined using a modified version of the Bliss independence reference model [79]. These values were used as the output variable in our models. Since a standard synergy metric does not currently exist and considering that different synergy metrics may lead to different conclusions, we opted to use the synergy metric defined by the original ALMANAC study instead of another metric based on a different reference model. This synergy metric has also been used as the output variable in other studies using ALMANAC data [19, 20, 40, 43].

Since ComboScore predictions should be independent of the order of the drugs in a given combination, we considered reverse drug order <*cell line*, *drugB*, *drugA*> triplets to be duplicates and the ComboScores for experiments involving the same cell line and same drug combination were averaged. The MDA-MB-468 cell line that is present in the original ALMANAC dataset was not considered in our analysis as it does not appear in the omics datasets we used in this study. The MDA-N cell line present in the omics datasets from CellMinerCDB was removed as it was not included in the ALMANAC project.

National Service Center (NSC) compound identifiers were used to map compounds to their respective Simplified Molecular-Input Line-Entry System (SMILES) strings using a compound structure-data file (SDF) file provided by NCI Developmental Therapeutics Program (DTP) for the ALMANAC dataset. For the compounds that were not successfully mapped using the previously described method, PubChemPy (version 1.0.4) was used to obtain canonical SMILES strings from PubChem [80] based on drug names. The SMILES strings were then preprocessed using the ChEMBL Structure Pipeline (version 1.0.0) [81], which removed salts and standardized the molecules according to ChEMBL-defined rules. The preprocessed SMILES were then used to compute different compound representations, depending on the type of architecture that was chosen for the drug subnetwork.

RNA-Seq gene expression data (log2(FPKM+1)) for the NCI-60 cell lines were also downloaded from CellMinerCDB [24]. Genes with constant expression values across all cell lines were removed. The expression dataset was then filtered using specific gene lists.

Besides the use of specific gene sets to reduce the number of features in the gene expression dataset, several other dimensionality reduction methods were also tested. UMAP was used to further reduce the dimensionality of the gene expression dataset filtered by protein coding genes (*expr_protein coding_* dataset). Embeddings with 50 components were generated using the UMAP Python package (version 0.5.1) [82]. Since UMAP can be used as a data transformer, the algorithm was fit on the training data to learn the latent space and then used to transform the training, validation and test sets into the learned latent space. The datasets were min-max scaled beforehand. A total of 20 neighbors were considered when learning the manifold structure of the data. Pearson correlation was used as the distance metric, and the minimum distance between two points in the embedding was kept at the default value of 0.1.

We also evaluated the use of WGCNA [83] as a dimensionality reduction technique for gene expression data. The WGCNA R package (version 1.69) was used to find a total of 136 gene co-expression modules. Module eigengenes, which summarize the expression of genes in each module, were then used as input features instead of the original gene expression values.

When preparing the gene expression (*expr_protein coding_*) data to be fed into 1D or 2D CNNs, two different methods were used to rearrange the genes so that their locations within the input tensors might be more biologically meaningful. A schematic representation of the methods is provided in S6 Fig. The first method sorted the genes according to their chromosome positions, an approach similar to the method described in [84]. The second method employed hierarchical clustering to find a new order for the genes, with the goal of placing genes with related expression patterns closer together within the input tensors. The clustering algorithm used Pearson correlation as the distance metric and complete linkage. The clustering approach that was used is agglomerative, successively merging smaller clusters into larger ones based on the distance between clusters. The resulting linkage matrix was reordered so that the distance between successive leaves would be minimized. Subsequently, a dendrogram was created based on the clustering results, and the positions of the leaves (genes) was used to reorder the gene expression features. The gene expression data were then reshaped into the required formats for 1D and 2D CNNs.

A mutation annotation format (MAF) file containing mutation data from whole-exome sequencing for NCI-60 cell lines was downloaded from cBioPortal [29, 30]. Silent mutations and mutations in non-coding regions were excluded. The remaining data were then binarized and summarized at the gene level, with ‘1’ indicating that a given cell line had at least one alteration in a particular gene and zero indicating the absence of mutations. To create the gene-level mutation dataset, the data were further filtered using a list of drug-gene interactions from DGIdb, leaving a total of 636 genes. To create the pathway-level dataset, a new binary event matrix was created, where a ‘1’ indicates that a given cell line had at least one alteration in a given pathway. Genes were mapped to pathways using Molecular Signatures Database (MSigDB) [85, 86] (version 7.2) gene sets derived from the Reactome [87] database, which have been filtered to reduce the redundancy between gene sets. The pathway-level mutation dataset had a total of 1,510 features.

Genomic Identification of Significant Targets in Cancer (GISTIC)2 putative CNVs for the NCI-60 cell lines were also obtained from cBioPortal [29, 30]. Only genes that were present in the drug-gene interactions list obtained from DGIdb were kept, leaving a total of 952 features.

The omics datasets were individually merged with the drug response dataset to create the final omics datasets containing the corresponding cell line data for each experiment. After splitting the expression dataset into training/validation/test sets, the features were scaled to a range between zero and one (min-max scaling), with the exception of the UMAP and WGCNA-transformed data, which were not scaled after the dimensionality reduction step.

The data were initially randomly split into three subsets—a training set comprising around 80% of the data (239,943 examples), and validation and test sets each corresponding to approximately 10% of the original data (30,007 and 30,001 examples, respectively).

### Models

The DL models were built using a multimodal approach, in which each data type has its own corresponding feature-encoding subnetwork. Several different architectures were tested for each of the feature-encoding subnetworks. The learned representations from each subnetwork were then concatenated into a single tensor before being fed into a final drug synergy prediction subnetwork. The prediction subnetwork was always entirely composed of fully connected layers, ending in a single output unit with a linear activation function, given that the prediction task was approached as a regression problem.

First, we built several baseline models: a random baseline model that always predicts the average ComboScore value of the training set, and baseline models where one-hot encoded identifiers were used instead of cell line features and/or drug features (*cell line_one hot_ + drugs_one hot_*, *cell line_one hot_ + drugs_ECFP4_*, and *expr_DGI_ + drugs_one hot_*).

We then built models trained on two different combinations of input data types:

1. *expr + drugA + drugB* models—use of RNA-Seq gene expression data (*expr*) and drug features for both drugs in a combination (*drugA*, *drugB*);
2. *expr + mut + cnv + drugA + drugB*—use of expression data (*expr*), mutation data (*mut*), copy number variation features (*cnv*) and drug features (*drugA*, *drugB*).

We began with type 1 models and tested the impact of the format of the transcriptomics data. We evaluated the influence of the type of architecture used to process the gene expression values (fully connected, 1D CNNs and 2D CNNs) and the influence of the arrangement and order of the genes (*chromosome order* vs. *clustering order*).

We then tested the extension of the gene sets, by using more comprehensive or more selective lists of genes. We also tested if using directly normalized expression values or compact representations that capture the variability and correlation showed any differences in performance. The following feature selection/dimensionality reduction methods were evaluated in this study:

- *expr_protein coding_*: the original dataset was filtered so that only protein coding genes were kept, leaving a total of 18,779 genes;
- *expr_landmark_*: a list of “landmark genes” as defined by the L1000 project was used to reduce the gene expression dataset to 978 genes that are considered to be representative of the transcriptome as a whole [88];
- *expr_DGI_*: DGI claims were obtained from DGIdb [89] (version 4.2.0) and then used to build a list of 994 DGI genes for the compounds screened in the ALMANAC project. 976 of these genes were present in the gene expression dataset;
- *expr_COSMIC_*: A list of 723 cancer driver genes was obtained from the COSMIC [90] (version 94) Cancer Gene Census [91]. After filtering, the gene expression dataset contained 713 genes;
- *expr_NCG_*: A different cancer-specific gene list containing 2,372 genes was obtained from the Network of Cancer Genes (NCG) (version 6.0) [92]. It includes genes identified in the COSMIC Cancer Gene Census, as well as other genes that have been implicated in cancer. After filtering, 2,362 genes remained in the expression dataset;
- Combined gene lists: *expr_DGI + landmark_* (1,815 features) and *expr_DGI + NCG_* (3,037 features);
- *expr_UMAP_*: UMAP [82] was used to capture the non-linear structure in the high-dimensional gene expression data, projecting it into a low-dimensional representation (50 components);
- *expr_WGCNA_*: WGCNA [83] was used to find network modules that capture the correlated expression of a set of genes. Module eigengenes summarize the expression of genes in each module and can be used to reduce the dimensionality of the gene expression data. The reduced expressed dataset had a total of 136 module eigengenes.

Next, we kept the expression-encoding subnetwork fixed and modified the drug-encoding subnetworks to assess the impact of different drug representations:

- *drugs_ECFP4_*: 1024-bit Morgan fingerprints with a radius of 2 (equivalent to ECFP4 fingerprints [93]) fed into fully connected drug subnetworks;
- *drugs_LayeredFP_*: 1024-bit layered fingerprints fed into fully connected drug subnetworks;
- *drugs_TextCNN_*: SMILES strings were tokenized and one-hot encoded, and then fed into TextCNN [94, 95] subnetworks;
- *drugs_GCN_*: using GCNs [96] for the drug subnetworks;
- *drugs_GAT_*: using GATs [97] for the drug subnetworks;
- *drugs_MTE_*: 512-dimensional Molecular Transformer Embeddings (MTEs) [98], fed into fully connected drug subnetworks.

Each drug in a combination had a separate feature-encoding subnetwork, with weights being shared between the two drug subnetworks.

Afterwards, we examined if additional data on mutations and CNVs would lead to improved predictive performance (type 2 models). We tested two different models: one trained on mutation data summarized at the gene level and CNVs, filtered to only include DGI genes (*expr_DGI_ + mut_DGI, gene-level_ + cnv_DGI_ + drugs_ECFP4_* model); a second model trained using mutations summarized at the pathway level and CNVs (*expr_DGI_ + mut_pathway-level_ + cnv_DGI_ + drugs_ECFP4_* model). When included, the mutation and CNV subnetworks were always fully connected.

All of the models were implemented using the Keras subpackage in Tensorflow [99] (version 2.2.0). The GCN and GAT subnetworks were built using graph layers implemented in the Spektral package [100] (version 1.0.7).

### Model hyperparameters and hyperparameter optimization

All models used the mean squared error (MSE) as the loss function, and the adaptive moment estimation (Adam) algorithm as the optimization method. Models were trained for a maximum of 500 epochs, using the EarlyStopping callback with a patience of 15 epochs to stop the learning process once the validation loss stopped improving. The mini-batch size was 64.

A selection of model hyperparameters, including the learning rate, the hidden layer activation function, the dropout rate and the L2 regularization penalty, as well as subnetwork-specific hyperparameters, were optimized for each combination of input data types and subnetwork architectures that we evaluated in this work. Hyperparameters were tuned using the validation set and the Bayesian Optimization with HyperBand (BOHB) algorithm [101] as implemented in Ray Tune (version 1.0.1). The hyperparameter search space was explored using Bayesian optimization [102] and the HyperBand [103] algorithm was used to stop underperforming trials early. A total of 50 configurations were evaluated for each model. The set of hyperparameters that minimized the validation MSE was considered the best configuration. More details on the hyperparameter search grids that were explored and the hyperparameter values that were chosen for each model are provided in S1 File.

### Model evaluation

After tuning the model hyperparameters, the models were evaluated on the independent test set. Model performance was evaluated using several scoring metrics, including the R^2^ and Spearman’s rank correlation coefficient. The scores were calculated over the entire test set.

In addition to comparing DL models with different omics and drug subnetworks, our models were also compared against non-DL models. Elastic net linear regression [104], SVR [105], and RF [106] models were implemented using the scikit-learn Python package (version 0.22.1) [107]. Since kernelized SVR does not scale well to larger datasets, we used the Nystroem method [108] to approximate the feature mappings of a RBF kernel before training a linear SVR on these approximations. An XGBoost [109] model was implemented using the xgboost Python package (version 1.4.0), and a LGBM [110] model was implemented using the lightgbm package (version 3.2.1). The ML models were trained on ECFP4 fingerprints and the gene expression values of DGI genes based on information from DGIdb. The input features were concatenated into a single dataset before training. Hyperparameters were optimized using Bayesian optimization.

### Heterogeneous ensemble

We developed a simple heterogeneous ensemble combining the top 10 DL models developed in this study and the RF, XGBoost and LGBM models. To obtain the ensemble prediction, the predictions from each of the individual models were simply averaged.

### Feature importance

The SHAP Python package (version 0.39.0) [49] was used to explain predictions made by the best DL models developed in this work. More specifically, we used Deep SHAP, which uses the Deep Learning Important FeaTures (DeepLIFT) [111] additive feature attribution method to approximate SHAP values. Feature importance was then determined based on these values. The most important fingerprint bits were visualized using drawing functions available in RDKit (version 2020.09.1.0).

A pre-ranked GSEA [62] was performed for test set example 11835 using the clusterProfiler R package (version 3.18.1) [112]. SHAP values were used to rank the genes prior to the analysis.

## Supporting information

S1 Fig Common mechanisms of joint action underlying the effects of known synergistic drug combinations. (TIF)

S2 Fig The different types of deep learning and machine learning models developed in this study. (TIF)

S3 Fig 2D depiction of the 20 most important ECFP4 bits overall, according to Fig 3. (TIF)

S4 Fig 2D depiction of the top 15 most important ECFP4 “ON” bits with a positive effect on the model output for drugA (Topotecan) in test set example 11835. (TIF)

S5 Fig 2D depiction of the top 15 most important ECFP4 “ON” bits with a positive effect on the model output for drugB (Gefitinib) in test set example 11835. (TIF)

S6 Fig A schematic representation of the 1D and 2D CNN gene expression-encoding subnetworks. (A) The use of chromosome positions of genes or hierarchical clustering to rearrange the gene expression features. (B) Reshaping data for the 1D CNN feature-encoding subnetwork. (C) Reshaping the data for the 2D CNN feature-encoding subnetwork. (TIF)

S1 Table Performance scores for all of the models developed in this study. (CSV)

S2 Table Top 20 features (ranked by Gini importance) for the random forest model. (CSV)

S3 Table Pre-ranked GSEA results. (CSV)

S1 File Hyperparameter search grids and selected hyperparameter values for each model. (PDF)
