# Supplementary figures and images for: A systematic evaluation of deep learning methods for the prediction of drug synergy in cancer
Source: PLoS Comput Biol. 2023 Mar 23;19(3):e1010200. doi: 10.1371/journal.pcbi.1010200 (PMC10072473; doi:10.1371/journal.pcbi.1010200)

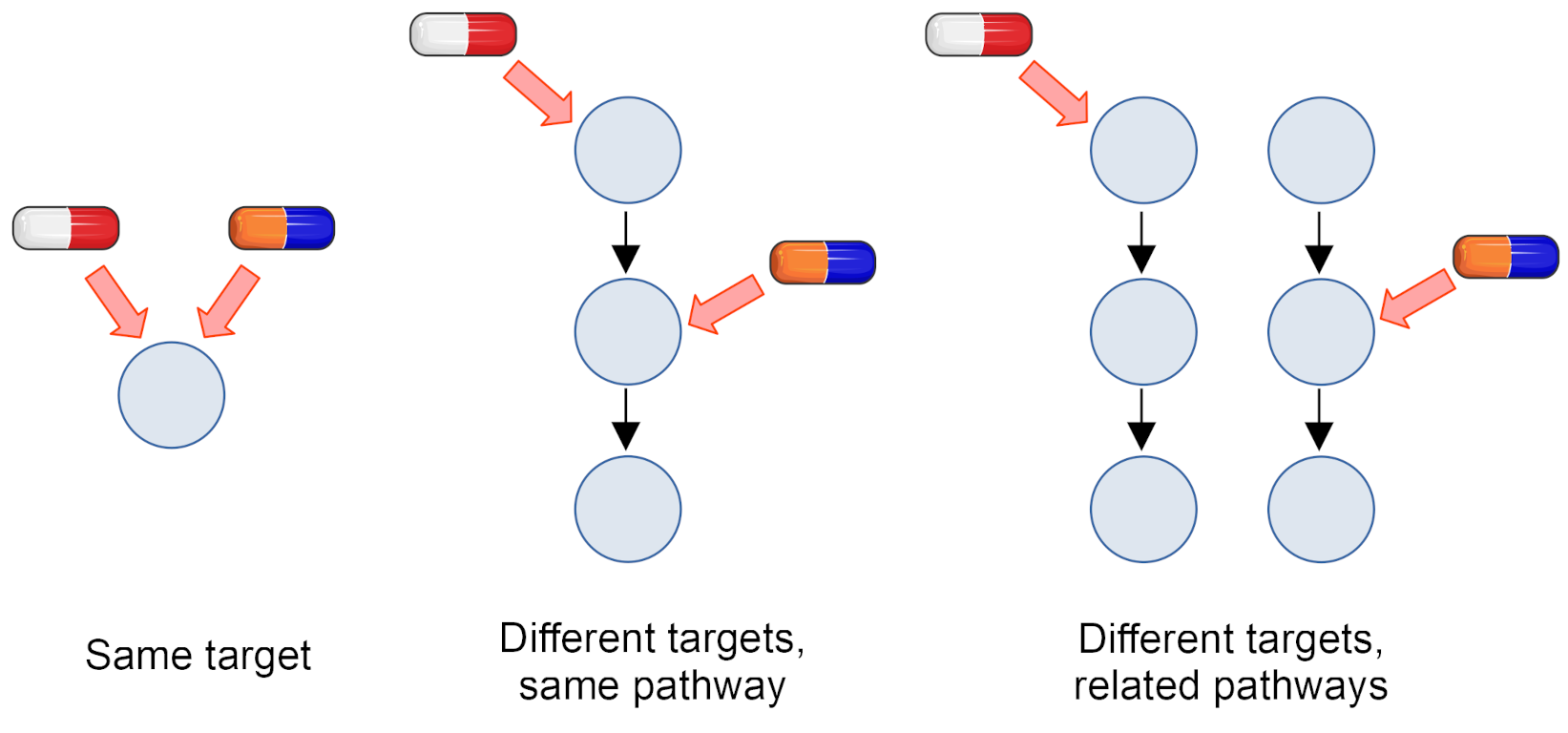

Supplement: S1 Fig — (TIF) [file pcbi.1010200.s001.tif]

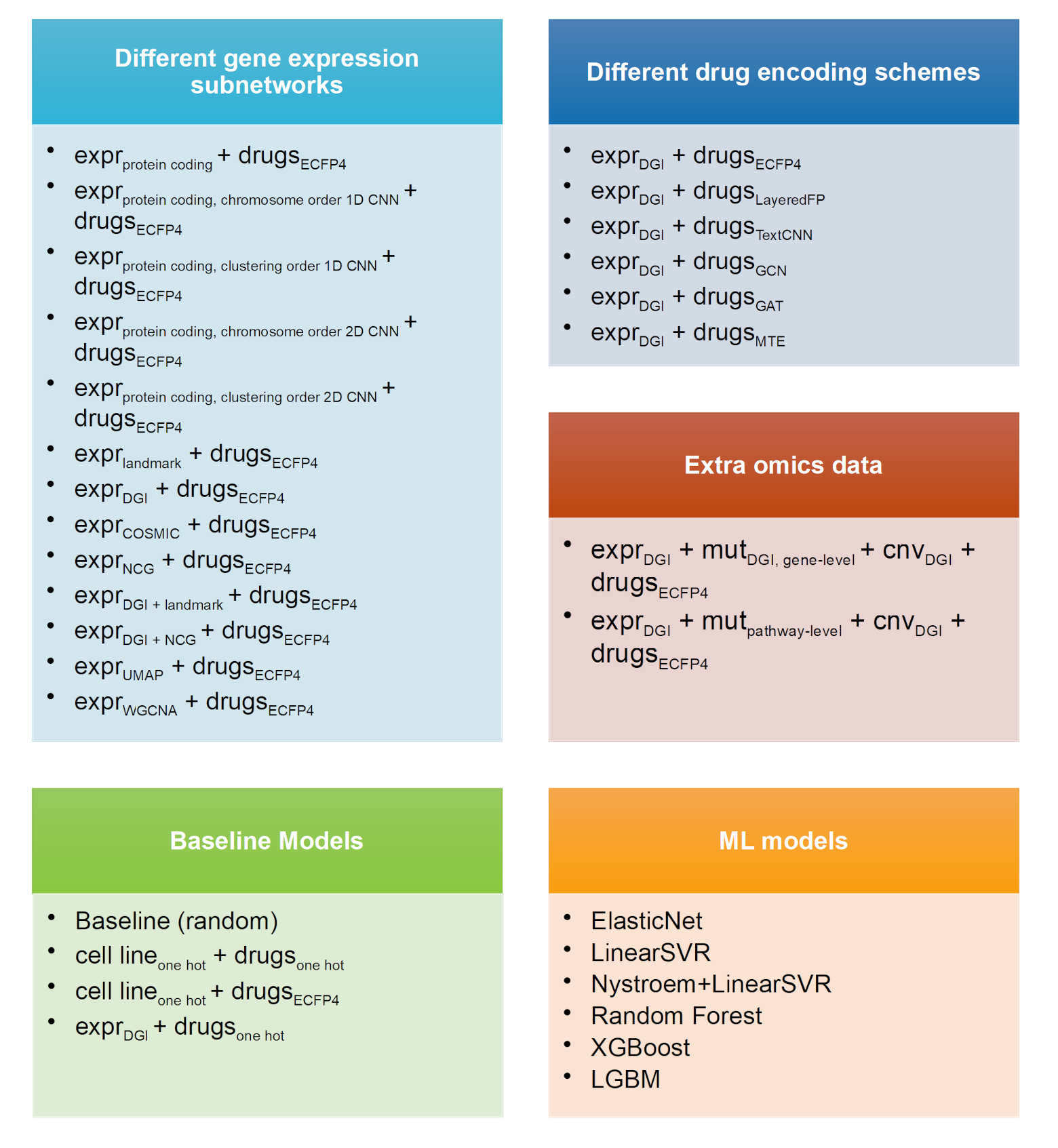

Supplement: S2 Fig — (TIF) [file pcbi.1010200.s002.tif]

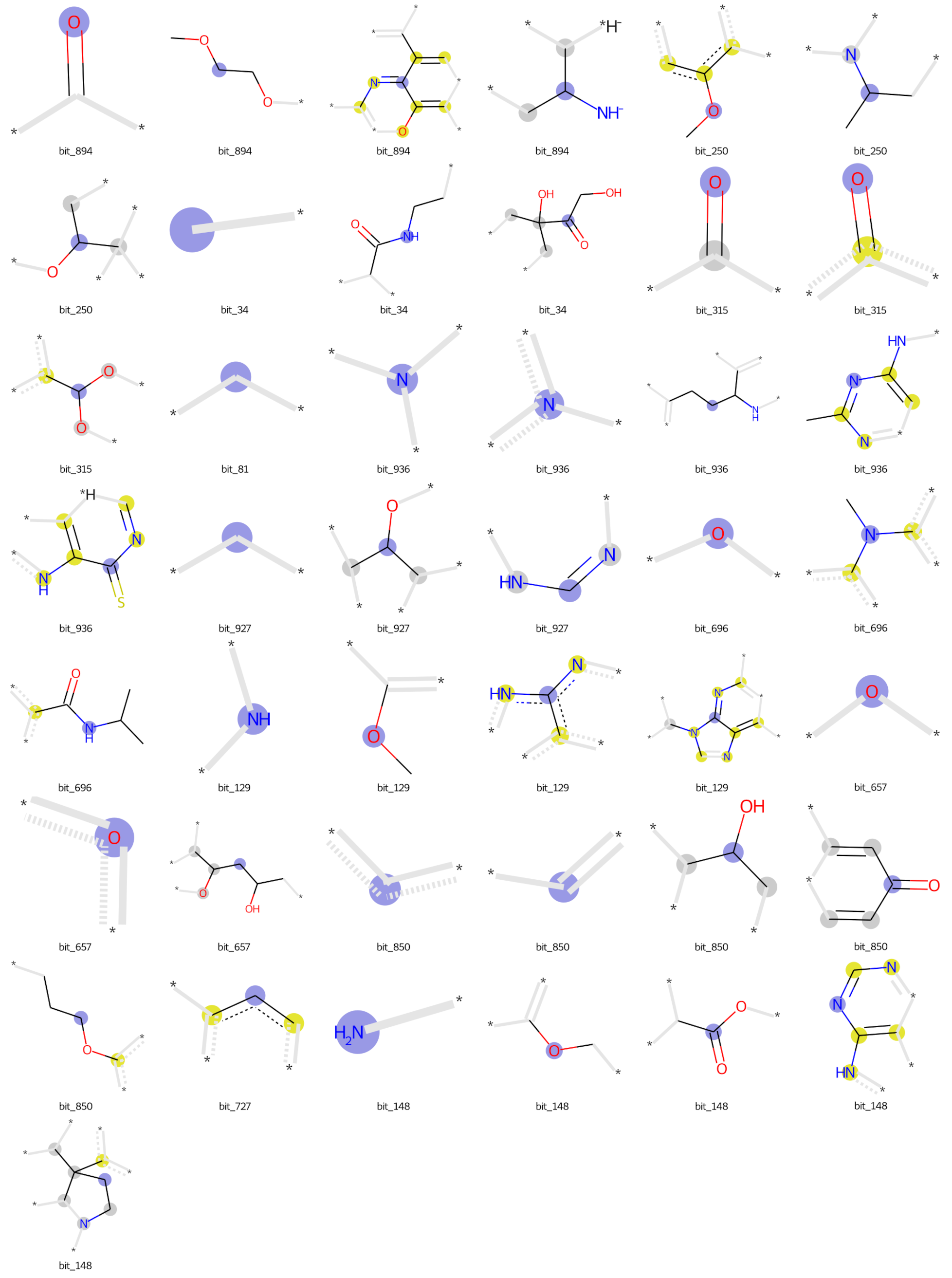

Supplement: S3 Fig — (TIF) [file pcbi.1010200.s003.tif]

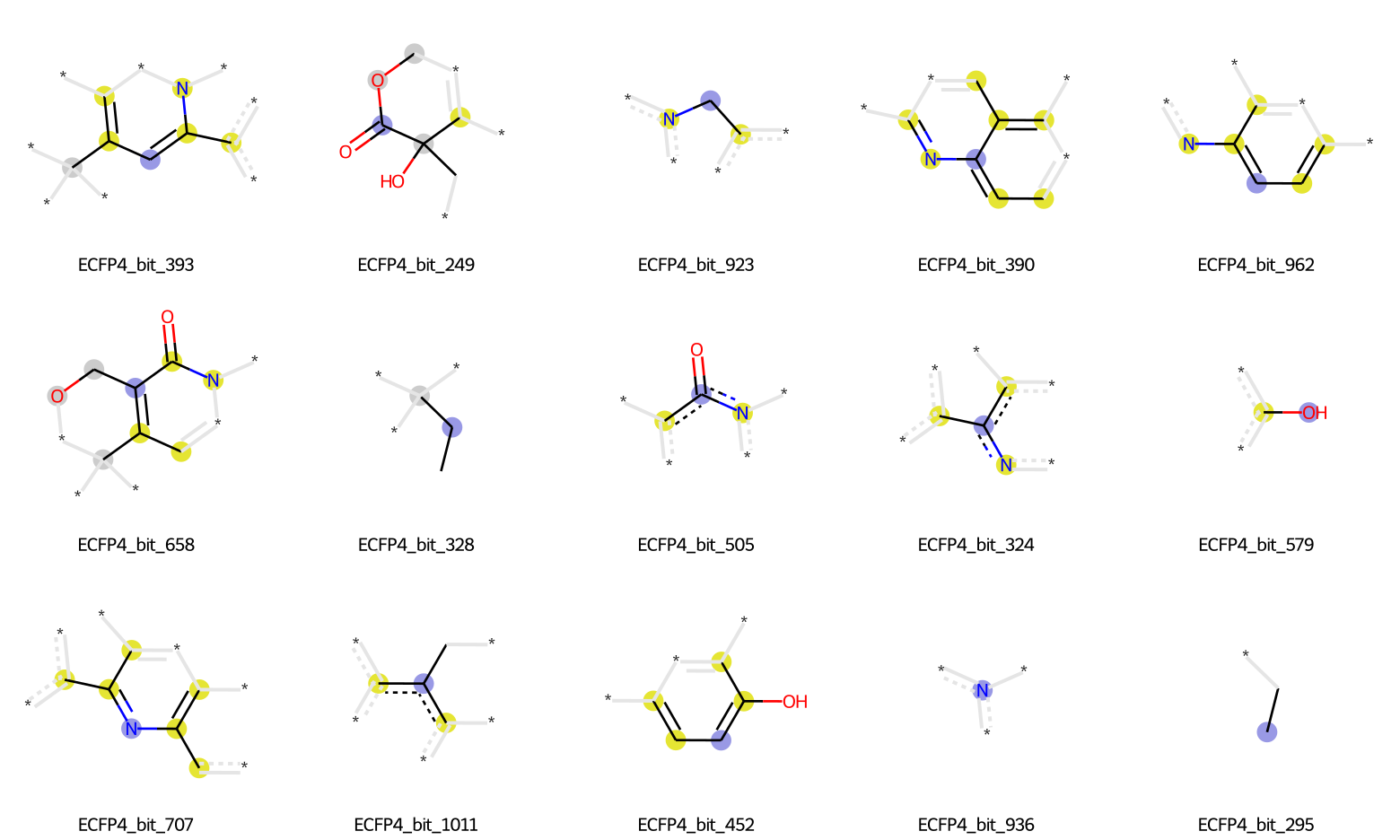

Supplement: S4 Fig — (TIF) [file pcbi.1010200.s004.tif]

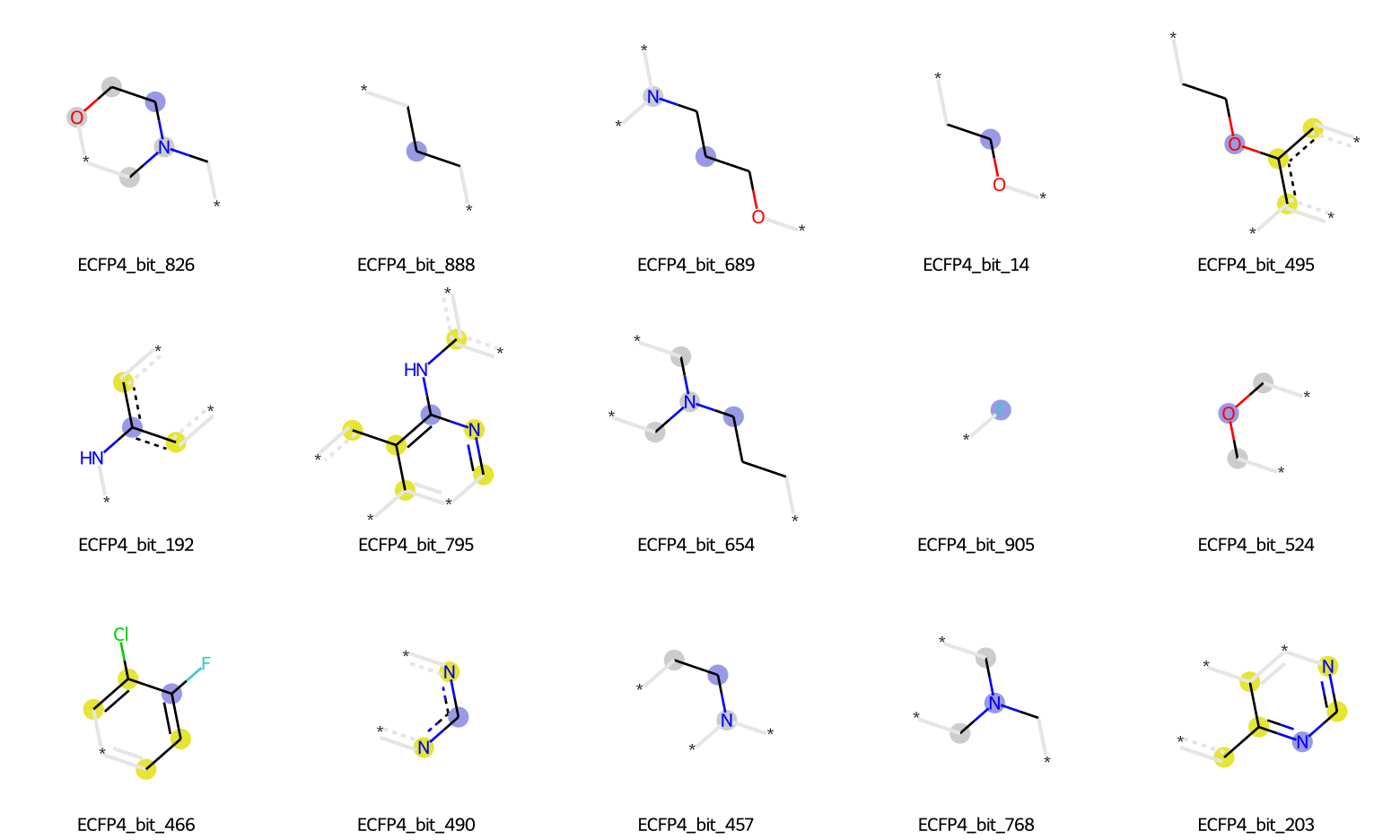

Supplement: S5 Fig — (TIF) [file pcbi.1010200.s005.tif]

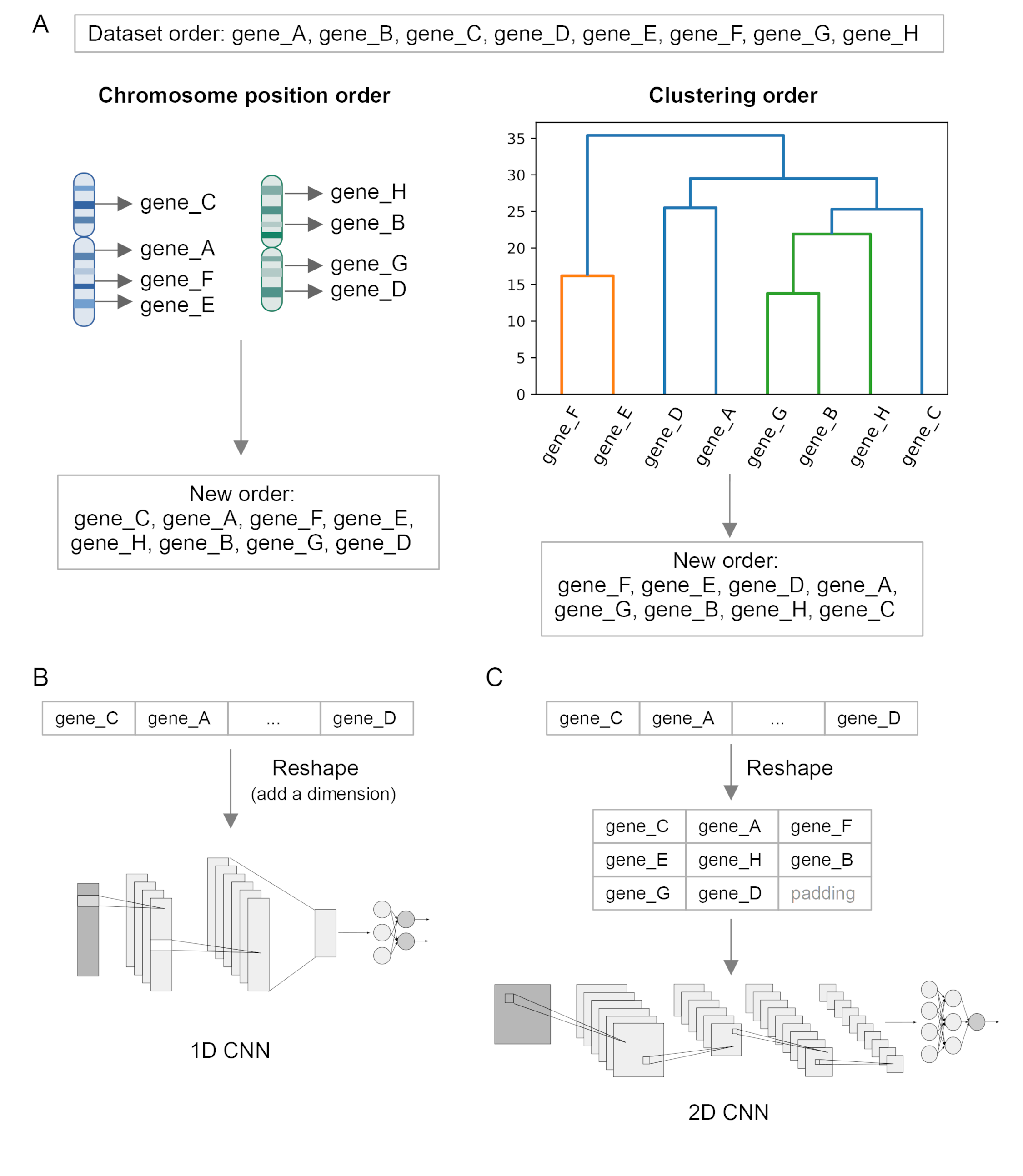

Supplement: S6 Fig — (TIF) [file pcbi.1010200.s006.tif]
