# Supplementary material for: A systematic evaluation of deep learning methods for the prediction of drug synergy in cancer
Source: PLoS Comput Biol. 2023 Mar 23;19(3):e1010200. doi: 10.1371/journal.pcbi.1010200 (PMC10072473; doi:10.1371/journal.pcbi.1010200)
Supplement: S1 File — (PDF) [file pcbi.1010200.s010.pdf]

Table A – Hyperparameter search grid for the *cell line*$_{one\ hot}$ + *drugs*$_{one\ hot}$ model.

Hyperparameter	Values tested
expr_hlayers_sizes	[64], [32], [16], [8], [4], [64, 32], [32, 16], [16, 8], [8, 4], [64, 32, 16], [32, 16, 8], [16, 8, 4]
drug_hlayers_sizes	[64], [32], [16], [8], [4], [64, 32], [32, 16], [16, 8], [8, 4], [64, 32, 16], [32, 16, 8], [16, 8, 4]
predictor_hlayers_sizes	[1024], [512], [256], [128], [64], [32], [1024, 512], [512, 256], [256, 128], [128, 64], [64, 32], [1024, 512, 256], [512, 256, 128], [256, 128, 64], [128, 64, 32], [1024, 1024], [512, 512], [256, 256], [128, 128], [64, 64], [32, 32], [1024, 1024, 1024], [512, 512, 512], [256, 256, 256], [128, 128, 128], [64, 64, 64], [32, 32, 32]
hidden_dropout	0.0, 0.1, 0.2, 0.3, 0.4, 0.5
hidden_activation	relu, leakyrelu, prelu
l2	0.00001, 0.0001, 0.001, 0.01, 0.1, 0
learn_rate	0.00001, 0.0001, 0.001, 0.01, 0.1

Table B – Hyperparameter search grid for the *cell line*$_{one\ hot}$ + *drugs*$_{ECFP4}$ model.

Hyperparameter	Values tested
expr_hlayers_sizes	[64], [32], [16], [8], [4], [64, 32], [32, 16], [16, 8], [8, 4], [64, 32, 16], [32, 16, 8], [16, 8, 4]
drug_hlayers_sizes	[1024], [512], [256], [128], [64], [1024, 512], [512, 256], [256, 128], [128, 64], [1024, 512, 256],[512, 256, 128], [256, 128, 64]
predictor_hlayers_sizes	[1024], [512], [256], [128], [64], [32], [1024, 512], [512, 256], [256, 128], [128, 64], [64, 32], [1024, 512, 256], [512, 256, 128], [256, 128, 64], [128, 64, 32], [1024, 1024], [512, 512], [256, 256], [128, 128], [64, 64], [32, 32], [1024, 1024, 1024], [512, 512, 512], [256, 256, 256], [128, 128, 128], [64, 64, 64], [32, 32, 32]
hidden_dropout	0.0, 0.1, 0.2, 0.3, 0.4, 0.5
hidden_activation	relu, leakyrelu, prelu
l2	0.00001, 0.0001, 0.001, 0.01, 0.1, 0
learn_rate	0.00001, 0.0001, 0.001, 0.01, 0.1

Table C – Hyperparameter search grid for the *$expr_{DGI}$* + *$drugs_{one\ hot}$* model.

Hyperparameter	Values tested
expr_hlayers_sizes	[1024], [512], [256], [128], [64], [32], [16], [1024, 512], [512, 256], [256, 128], [128, 64], [64, 32], [32, 16], [1024, 512, 256], [512, 256, 128], [256, 128, 64], [128, 64, 32], [64, 32, 16]
drug_hlayers_sizes	[64], [32], [16], [8], [4], [64, 32], [32, 16], [16, 8], [8, 4], [64, 32, 16], [32, 16, 8], [16, 8, 4]
predictor_hlayers_sizes	[1024], [512], [256], [128], [64], [32], [1024, 512], [512, 256], [256, 128], [128, 64], [64, 32], [1024, 512, 256], [512, 256, 128], [256, 128, 64], [128, 64, 32], [1024, 1024], [512, 512], [256, 256], [128, 128], [64, 64], [32, 32], [1024, 1024, 1024], [512, 512, 512], [256, 256, 256], [128, 128, 128], [64, 64, 64], [32, 32, 32]
hidden_dropout	0.0, 0.1, 0.2, 0.3, 0.4, 0.5
hidden_activation	relu, leakyrelu, prelu
l2	0.00001, 0.0001, 0.001, 0.01, 0.1, 0
learn_rate	0.00001, 0.0001, 0.001, 0.01, 0.1

Table D – Hyperparameter search grid for the *$expr_{protein\ coding}$* + *$drugs_{ECFP4}$* model.

Hyperparameter	Values tested
expr_hlayers_sizes	[8192], [4096], [2048], [1024], [512], [256], [128], [8192, 4096], [4096, 2048], [2048, 1024], [1024, 512], [512, 256], [256, 128], [8192, 4096, 2048], [4096, 2048, 1024], [2048, 1024, 512], [1024, 512, 256], [512, 256, 128]
drug_hlayers_sizes	[1024], [512], [256], [128], [64], [1024, 512], [512, 256], [256, 128], [128, 64], [1024, 512, 256], [512, 256, 128], [256, 128, 64]
predictor_hlayers_sizes	[4096], [2048], [1024], [512], [256], [128], [64], [4096, 2048], [2048, 1024], [1024, 512], [512, 256], [256, 128], [128, 64], [4096, 2048, 1024], [2048, 1024, 512], [1024, 512, 256], [512, 256, 128], [256, 128, 64], [4096, 4096], [2048, 2048], [1024, 1024], [512, 512], [256, 256], [128, 128], [64, 64], [4096, 4096, 4096], [2048, 2048, 2048], [1024, 1024, 1024], [512, 512, 512], [256, 256, 256], [128, 128, 128], [64, 64, 64]
hidden_dropout	0.0, 0.1, 0.2, 0.3, 0.4, 0.5
hidden_activation	relu, leakyrelu, prelu
l2	0.00001, 0.0001, 0.001, 0.01, 0.1, 0
learn_rate	0.00001, 0.0001, 0.001, 0.01, 0.1

Table E – Hyperparameter search grid for the *expr*$_{protein\ coding,\ chromosome\ order\ 1D\ CNN}$ + *drugs*$_{ECFP4}$ and *expr*$_{protein\ coding,\ clustering\ order\ 1D\ CNN}$ + *drugs*$_{ECFP4}$ models.

Hyperparameter	Values tested
expr_num_filters	[16], [32], [64], [16, 16], [32, 32], [64, 64], [16, 32], [32, 64]
expr_kernel_sizes *	[3, 3], [5, 5], [10, 10], [20, 20]
expr_pool_size	5, 10
expr_batchnorm	True, False
drug_hlayers_sizes	[1024], [512], [256], [128], [64], [1024, 512], [512, 256], [256, 128], [128, 64], [1024, 512, 256], [512, 256, 128], [256, 128, 64]
predictor_hlayers_sizes	[4096], [2048], [1024], [512], [256], [128], [64], [4096, 2048], [2048, 1024], [1024, 512], [512, 256], [256, 128], [128, 64], [4096, 2048, 1024], [2048, 1024, 512], [1024, 512, 256], [512, 256, 128], [256, 128, 64], [4096, 4096], [2048, 2048], [1024, 1024], [512, 512], [256, 256], [128, 128], [64, 64], [4096, 4096, 4096], [2048, 2048, 2048], [1024, 1024, 1024], [512, 512, 512], [256, 256, 256], [128, 128, 128], [64, 64, 64]
hidden_dropout	0.0, 0.1, 0.2, 0.3, 0.4, 0.5
hidden_activation	relu, leakyrelu, prelu
l2	0.00001, 0.0001, 0.001, 0.01, 0.1, 0
learn_rate	0.00001, 0.0001, 0.001, 0.01, 0.1

* The actual number of values that will be used from the expr_kernel_sizes list will depend on the length of expr_num_filters (if the length of expr_num_filters is 1, only the first value will be used from the provided expr_kernel_sizes list)

Table F – Hyperparameter search grid for the *expr*$_{protein\ coding,\ chromosome\ order\ 2D\ CNN}$ + *drugs*$_{ECFP4}$ and the *expr*$_{protein\ coding,\ clustering\ order\ 2D\ CNN}$ + *drugs*$_{ECFP4}$ models.

Hyperparameter	Values tested
expr_num_filters	[16], [32], [64], [128], [16, 32], [32, 64], [64, 128], [16, 32, 64], [32, 64, 128]
expr_kernel_size	(3, 3), (5, 5)
expr_pool_size	(2,2)
expr_batchnorm	True, False
drug_hlayers_sizes	[1024], [512], [256], [128], [64], [1024, 512], [512, 256], [256, 128], [128, 64], [1024, 512, 256], [512, 256, 128], [256, 128, 64]
predictor_hlayers_sizes	[4096], [2048], [1024], [512], [256], [128], [64], [4096, 2048], [2048, 1024], [1024, 512], [512, 256], [256, 128], [128, 64], [4096, 2048, 1024], [2048, 1024, 512], [1024, 512, 256], [512, 256, 128], [256, 128, 64], [4096, 4096], [2048, 2048], [1024, 1024], [512, 512], [256, 256], [128, 128], [64, 64], [4096, 4096, 4096], [2048, 2048, 2048], [1024, 1024, 1024], [512, 512, 512], [256, 256, 256], [128, 128, 128], [64, 64, 64]
hidden_dropout	0.0, 0.1, 0.2, 0.3, 0.4, 0.5
hidden_activation	relu, leakyrelu, prelu
l2	0.00001, 0.0001, 0.001, 0.01, 0.1, 0
learn_rate	0.00001, 0.0001, 0.001, 0.01, 0.1

Table G – Hyperparameter search grid for the *expr$_{landmark}$* + *drugs$_{ECFP4}$* model.

Hyperparameter	Values tested
expr_hlayers_sizes	[1024], [512], [256], [128], [64], [32], [16], [1024, 512], [512, 256], [256, 128], [128, 64], [64, 32], [32, 16], [1024, 512, 256], [512, 256, 128], [256, 128, 64], [128, 64, 32], [64, 32, 16]
drug_hlayers_sizes	[1024], [512], [256], [128], [64], [1024, 512], [512, 256], [256, 128], [128, 64], [1024, 512, 256], [512, 256, 128], [256, 128, 64]
predictor_hlayers_sizes	[2048], [1024], [512], [256], [128], [64], [2048, 1024], [1024, 512], [512, 256], [256, 128], [128, 64], [2048, 1024, 512], [1024, 512, 256], [512, 256, 128], [256, 128, 64], [2048, 2048], [1024, 1024], [512, 512], [256, 256], [128, 128], [64, 64], [2048, 2048, 2048], [1024, 1024, 1024], [512, 512, 512], [256, 256, 256], [128, 128, 128], [64, 64, 64]
hidden_dropout	0.0, 0.1, 0.2, 0.3, 0.4, 0.5
hidden_activation	relu, leakyrelu, prelu
l2	0.00001, 0.0001, 0.001, 0.01, 0.1, 0
learn_rate	0.00001, 0.0001, 0.001, 0.01, 0.1

Table H – Hyperparameter search grid for the *expr*$_{DGI}$ + *drugs*$_{ECFP4}$ model.

Hyperparameter	Values tested
expr_hlayers_sizes	[1024], [512], [256], [128], [64], [32], [16], [1024, 512], [512, 256], [256, 128], [128, 64], [64, 32], [32, 16], [1024, 512, 256], [512, 256, 128], [256, 128, 64], [128, 64, 32], [64, 32, 16]
drug_hlayers_sizes	[1024], [512], [256], [128], [64], [1024, 512], [512, 256], [256, 128], [128, 64], [1024, 512, 256], [512, 256, 128], [256, 128, 64]
predictor_hlayers_sizes	[2048], [1024], [512], [256], [128], [64], [2048, 1024], [1024, 512], [512, 256], [256, 128], [128, 64], [2048, 1024, 512], [1024, 512, 256], [512, 256, 128], [256, 128, 64], [2048, 2048], [1024, 1024], [512, 512], [256, 256], [128, 128], [64, 64], [2048, 2048, 2048], [1024, 1024, 1024], [512, 512, 512], [256, 256, 256], [128, 128, 128], [64, 64, 64]
hidden_dropout	0.0, 0.1, 0.2, 0.3, 0.4, 0.5
hidden_activation	relu, leakyrelu, prelu
l2	0.00001, 0.0001, 0.001, 0.01, 0.1, 0
learn_rate	0.00001, 0.0001, 0.001, 0.01, 0.1

Table I – Hyperparameter search grid for the *expr*$_{COSMIC}$ + *drugs*$_{ECFP4}$ model.

Hyperparameter	Values tested
expr_hlayers_sizes	[512], [256], [128], [64], [32], [16], [8], [512, 256], [256, 128], [128, 64], [64, 32], [32, 16], [16, 8], [512, 256, 128], [256, 128, 64], [128, 64, 32], [64, 32, 16], [32, 16, 8]
drug_hlayers_sizes	[1024], [512], [256], [128], [64], [1024, 512], [512, 256], [256, 128], [128, 64], [1024, 512, 256], [512, 256, 128], [256, 128, 64]
predictor_hlayers_sizes	[2048], [1024], [512], [256], [128], [64], [2048, 1024], [1024, 512], [512, 256], [256, 128], [128, 64], [2048, 1024, 512], [1024, 512, 256], [512, 256, 128], [256, 128, 64], [2048, 2048], [1024, 1024], [512, 512], [256, 256], [128, 128], [64, 64], [2048, 2048, 2048], [1024, 1024, 1024], [512, 512, 512], [256, 256, 256], [128, 128, 128], [64, 64, 64]
hidden_dropout	0.0, 0.1, 0.2, 0.3, 0.4, 0.5
hidden_activation	relu, leakyrelu, prelu
l2	0.00001, 0.0001, 0.001, 0.01, 0.1, 0
learn_rate	0.00001, 0.0001, 0.001, 0.01, 0.1

Table J – Hyperparameter search grid for the *expr*$_{NCG}$ + *drugs*$_{ECFP4}$ model.

Hyperparameter	Values tested
expr_hlayers_sizes	[2048, [1024], [512], [256], [128], [64], [32], [2048, 1024], [1024, 512], [512, 256], [256, 128], [128, 64], [64, 32], [2048, 1024, 512], [1024, 512, 256], [512, 256, 128], [256, 128, 64], [128, 64, 32]
drug_hlayers_sizes	[1024], [512], [256], [128], [64], [1024, 512], [512, 256], [256, 128], [128, 64], [1024, 512, 256], [512, 256, 128], [256, 128, 64]
predictor_hlayers_sizes	[2048], [1024], [512], [256], [128], [64], [2048, 1024], [1024, 512], [512, 256], [256, 128], [128, 64], [2048, 1024, 512], [1024, 512, 256], [512, 256, 128], [256, 128, 64], [2048, 2048], [1024, 1024], [512, 512], [256, 256], [128, 128], [64, 64], [2048, 2048, 2048], [1024, 1024, 1024], [512, 512, 512], [256, 256, 256], [128, 128, 128], [64, 64, 64]
hidden_dropout	0.0, 0.1, 0.2, 0.3, 0.4, 0.5
hidden_activation	relu, leakyrelu, prelu
l2	0.00001, 0.0001, 0.001, 0.01, 0.1, 0
learn_rate	0.00001, 0.0001, 0.001, 0.01, 0.1

Table K – Hyperparameter search grid for the *expr*$_{DGI + landmark}$ + *drugs*$_{ECFP4}$ model.

Hyperparameter	Values tested
expr_hlayers_sizes	[1024], [512], [256], [128], [64], [32], [16], [1024, 512], [512, 256], [256, 128], [128, 64], [64, 32], [32, 16], [1024, 512, 256], [512, 256, 128], [256, 128, 64], [128, 64, 32], [64, 32, 16]
drug_hlayers_sizes	[1024], [512], [256], [128], [64], [1024, 512], [512, 256], [256, 128], [128, 64], [1024, 512, 256], [512, 256, 128], [256, 128, 64]
predictor_hlayers_sizes	[2048], [1024], [512], [256], [128], [64], [2048, 1024], [1024, 512], [512, 256], [256, 128], [128, 64], [2048, 1024, 512], [1024, 512, 256], [512, 256, 128], [256, 128, 64], [2048, 2048], [1024, 1024], [512, 512], [256, 256], [128, 128], [64, 64], [2048, 2048, 2048], [1024, 1024, 1024], [512, 512, 512], [256, 256, 256], [128, 128, 128], [64, 64, 64]
hidden_dropout	0.0, 0.1, 0.2, 0.3, 0.4, 0.5
hidden_activation	relu, leakyrelu, prelu
l2	0.00001, 0.0001, 0.001, 0.01, 0.1, 0
learn_rate	0.00001, 0.0001, 0.001, 0.01, 0.1

Table L – Hyperparameter search grid for the *expr*$_{DGI + NCG}$ + *drugs*$_{ECFP4}$ model.

Hyperparameter	Values tested
expr_hlayers_sizes	[2048], [1024], [512], [256], [128], [64], [32], [2048, 1024], [1024, 512], [512, 256], [256, 128], [128, 64], [64, 32], [2048, 1024, 512], [1024, 512, 256], [512, 256, 128], [256, 128, 64],[128, 64, 32]
drug_hlayers_sizes	[1024], [512], [256], [128], [64], [1024, 512], [512, 256], [256, 128], [128, 64], [1024, 512, 256], [512, 256, 128], [256, 128, 64]
predictor_hlayers_sizes	[2048], [1024], [512], [256], [128], [64], [2048, 1024], [1024, 512], [512, 256], [256, 128], [128, 64], [2048, 1024, 512], [1024, 512, 256], [512, 256, 128], [256, 128, 64], [2048, 2048], [1024, 1024], [512, 512], [256, 256], [128, 128], [64, 64], [2048, 2048, 2048], [1024, 1024, 1024], [512, 512, 512], [256, 256, 256], [128, 128, 128], [64, 64, 64]
hidden_dropout	0.0, 0.1, 0.2, 0.3, 0.4, 0.5
hidden_activation	relu, leakyrelu, prelu
l2	0.00001, 0.0001, 0.001, 0.01, 0.1, 0
learn_rate	0.00001, 0.0001, 0.001, 0.01, 0.1

Table M – Hyperparameter search grid for the *expr*$_{UMAP}$ + *drugs*$_{ECFP4}$ model.

Hyperparameter	Values tested
expr_hlayers_sizes	[64], [32], [16], [8], [4], [64, 32], [32, 16], [16, 8], [8, 4], [64, 32, 16], [32, 16, 8], [16, 8, 4]
drug_hlayers_sizes	[1024], [512], [256], [128], [64], [1024, 512], [512, 256], [256, 128], [128, 64], [1024, 512, 256], [512, 256, 128], [256, 128, 64]
predictor_hlayers_sizes	[1024], [512], [256], [128], [64], [32], [1024, 512], [512, 256], [256, 128], [128, 64], [64, 32], [1024, 512, 256], [512, 256, 128], [256, 128, 64], [128, 64, 32], [1024, 1024], [512, 512], [256, 256] [128, 128], [64, 64], [32, 32], [1024, 1024, 1024], [512, 512, 512], [256, 256, 256], [128, 128, 128], [64, 64, 64], [32, 32, 32]
hidden_dropout	0.0, 0.1, 0.2, 0.3, 0.4, 0.5
hidden_activation	relu, leakyrelu, prelu
l2	0.00001, 0.0001, 0.001, 0.01, 0.1, 0
learn_rate	0.00001, 0.0001, 0.001, 0.01, 0.1

Table N – Hyperparameter search grid for the *expr*$_{WGCNA}$ + *drugs*$_{ECFP4}$ model.

Hyperparameter	Values tested
expr_hlayers_sizes	[128], [64], [32], [16], [8], [128, 64], [64, 32], [32, 16], [16, 8], [128, 64, 32], [64, 32, 16], [32, 16, 8]
drug_hlayers_sizes	[1024], [512], [256], [128], [64], [1024, 512], [512, 256], [256, 128], [128, 64], [1024, 512, 256], [512, 256, 128], [256, 128, 64]
predictor_hlayers_sizes	[1024], [512], [256], [128], [64], [32], [1024, 512], [512, 256], [256, 128], [128, 64], [64, 32], [1024, 512, 256], [512, 256, 128], [256, 128, 64], [128, 64, 32], [1024, 1024], [512, 512], [256, 256], [128, 128], [64, 64], [32, 32], [1024, 1024, 1024], [512, 512, 512], [256, 256, 256], [128, 128, 128], [64, 64, 64], [32, 32, 32]
hidden_dropout	0.0, 0.1, 0.2, 0.3, 0.4, 0.5
hidden_activation	relu, leakyrelu, prelu
l2	0.00001, 0.0001, 0.001, 0.01, 0.1, 0
learn_rate	0.00001, 0.0001, 0.001, 0.01, 0.1

Table O – Hyperparameter search grid for the *expr*$_{DGI}$ + *drugs*$_{LayeredFP}$ model.

Hyperparameter	Values tested
expr_hlayers_sizes	[1024], [512], [256], [128], [64], [32], [16], [1024, 512], [512, 256], [256, 128], [128, 64], [64, 32], [32, 16], [1024, 512, 256], [512, 256, 128], [256, 128, 64], [128, 64, 32], [64, 32, 16]
drug_hlayers_sizes	[1024], [512], [256], [128], [64], [1024, 512], [512, 256], [256, 128], [128, 64], [1024, 512, 256], [512, 256, 128], [256, 128, 64]
predictor_hlayers_sizes	[2048], [1024], [512], [256], [128], [64], [2048, 1024], [1024, 512], [512, 256], [256, 128], [128, 64], [2048, 1024, 512], [1024, 512, 256], [512, 256, 128], [256, 128, 64], [2048, 2048], [1024, 1024], [512, 512], [256, 256], [128, 128], [64, 64], [2048, 2048, 2048], [1024, 1024, 1024], [512, 512, 512], [256, 256, 256], [128, 128, 128], [64, 64, 64]
hidden_dropout	0.0, 0.1, 0.2, 0.3, 0.4, 0.5
hidden_activation	relu, leakyrelu, prelu
l2	0.00001, 0.0001, 0.001, 0.01, 0.1, 0
learn_rate	0.00001, 0.0001, 0.001, 0.01, 0.1

Table P – Hyperparameter search grid for the *expr*$_{DGI}$ + *drugs*$_{TextCNN}$ model.

Hyperparameter	Values tested
expr_hlayers_sizes	[1024], [512], [256], [128], [64], [32], [16], [1024, 512], [512, 256], [256, 128], [128, 64], [64, 32], [32, 16], [1024, 512, 256], [512, 256, 128], [256, 128, 64], [128, 64, 32], [64, 32, 16]
drug_dropout	0.0, 0.1, 0.2, 0.3, 0.4, 0.5
drug_kernel_sizes	[1, 2, 3, 4, 5, 6, 7, 8, 9, 10, 15, 20], [1, 2, 3, 4, 5, 7, 10, 15], [3, 4, 5, 7, 10, 15], [3, 4, 5, 7, 10], [3, 4, 5, 7], [3, 4, 5], [3, 5, 7]
drug_n_embedding	32, 64, 75
drug_num_filters *	[100, 200, 200, 200, 200, 100, 100, 100, 100, 100, 160, 160], [32, 32, 32, 32, 64, 64, 64, 64, 128, 128, 128, 128], [128, 128, 128, 128, 64, 64, 64, 64, 32, 32, 32, 32]
predictor_hlayers_sizes	[2048], [1024], [512], [256], [128], [64], [2048, 1024], [1024, 512], [512, 256], [256, 128], [128, 64], [2048, 1024, 512], [1024, 512, 256], [512, 256, 128], [256, 128, 64], [2048, 2048], [1024, 1024], [512, 512], [256, 256], [128, 128], [64, 64], [2048, 2048, 2048], [1024, 1024, 1024], [512, 512, 512], [256, 256, 256], [128, 128, 128], [64, 64, 64]
hidden_dropout	0.0, 0.1, 0.2, 0.3, 0.4, 0.5
hidden_activation	relu, leakyrelu, prelu
l2	0.00001, 0.0001, 0.001, 0.01, 0.1, 0
learn_rate	0.00001, 0.0001, 0.001, 0.01, 0.1

* The actual number of values that will be used from the drug_num_filters list will depend on the length of drug_kernel_sizes (if the length of drug_kernel_sizes is 3, only the first 3 values will be used from the provided drug_num_filters list)

Table Q – Hyperparameter search grid for the *expr*$_{DGI}$ + *drugs*$_{GCN}$ model.

Hyperparameter	Values tested
expr_hlayers_sizes	[1024], [512], [256], [128], [64], [32], [16], [1024, 512], [512, 256], [256, 128], [128, 64], [64, 32], [32, 16], [1024, 512, 256], [512, 256, 128], [256, 128, 64], [128, 64, 32], [64, 32, 16]
drug_gcn_layers	[32, 32], [64, 64], [128, 128], [256, 256], [32, 32, 32], [64, 64, 64], [128, 128, 128], [256, 256, 256], [32, 32, 32, 32], [64, 64, 64, 64], [128, 128, 128, 128], [256, 256, 256, 256]
drug_residual_connection	True, False
drug_dropout	0.0, 0.1, 0.2, 0.3, 0.4, 0.5
predictor_hlayers_sizes	[2048], [1024], [512], [256], [128], [64], [2048, 1024], [1024, 512], [512, 256], [256, 128], [128, 64], [2048, 1024, 512], [1024, 512, 256], [512, 256, 128], [256, 128, 64], [2048, 2048], [1024, 1024], [512, 512], [256, 256], [128, 128], [64, 64], [2048, 2048, 2048], [1024, 1024, 1024], [512, 512, 512], [256, 256, 256], [128, 128, 128], [64, 64, 64]
hidden_dropout	0.0, 0.1, 0.2, 0.3, 0.4, 0.5
hidden_activation	relu, leakyrelu, prelu
l2	0.00001, 0.0001, 0.001, 0.01, 0.1, 0
learn_rate	0.00001, 0.0001, 0.001, 0.01, 0.1

Table R – Hyperparameter search grid for the $\textit{expr}_{DGI}$ + $\textit{drugs}_{GAT}$ model.

Hyperparameter	Values tested
expr_hlayers_sizes	[1024], [512], [256], [128], [64], [32], [16], [1024, 512], [512, 256], [256, 128], [128, 64], [64, 32], [32, 16], [1024, 512, 256], [512, 256, 128], [256, 128, 64], [128, 64, 32], [64, 32, 16]
drug_gat_layers	[8, 8], [16, 16], [32, 32], [64, 64], [128, 128], [8, 8, 8], [16, 16, 16], [32, 32, 32], [64, 64, 64], [128, 128, 128]
drug_num_attention_heads	4, 6, 8
drug_concat_heads	True, False
drug_residual_connection	True, False
drug_dropout	0.0, 0.1, 0.2, 0.3, 0.4, 0.5
predictor_hlayers_sizes	[2048], [1024], [512], [256], [128], [64], [2048, 1024], [1024, 512], [512, 256], [256, 128], [128, 64], [2048, 1024, 512], [1024, 512, 256], [512, 256, 128], [256, 128, 64], [2048, 2048], [1024, 1024], [512, 512], [256, 256], [128, 128], [64, 64], [2048, 2048, 2048], [1024, 1024, 1024], [512, 512, 512], [256, 256, 256], [128, 128, 128], [64, 64, 64]
hidden_dropout	0.0, 0.1, 0.2, 0.3, 0.4, 0.5
hidden_activation	relu, leakyrelu, prelu
l2	0.00001, 0.0001, 0.001, 0.01, 0.1, 0
learn_rate	0.00001, 0.0001, 0.001, 0.01, 0.1

Table S – Hyperparameter search grid for the *expr*$_{DGI}$ + *drugs*$_{MTE}$ model.

Hyperparameter	Values tested
expr_hlayers_sizes	[1024], [512], [256], [128], [64], [32], [16], [1024, 512], [512, 256], [256, 128], [128, 64], [64, 32], [32, 16], [1024, 512, 256], [512, 256, 128], [256, 128, 64], [128, 64, 32], [64, 32, 16]
drug_hlayers_sizes	[512], [256], [128], [64], [32], [512, 256], [256, 128], [128, 64], [64, 32], [512, 256, 128], [256, 128, 64], [128, 64, 32]
predictor_hlayers_sizes	[2048], [1024], [512], [256], [128], [64], [2048, 1024], [1024, 512], [512, 256], [256, 128], [128, 64], [2048, 1024, 512], [1024, 512, 256], [512, 256, 128], [256, 128, 64], [2048, 2048], [1024, 1024], [512, 512], [256, 256], [128, 128], [64, 64], [2048, 2048, 2048], [1024, 1024, 1024], [512, 512, 512], [256, 256, 256], [128, 128, 128], [64, 64, 64]
hidden_dropout	0.0, 0.1, 0.2, 0.3, 0.4, 0.5
hidden_activation	relu, leakyrelu, prelu
l2	0.00001, 0.0001, 0.001, 0.01, 0.1, 0
learn_rate	0.00001, 0.0001, 0.001, 0.01, 0.1

Table T – Hyperparameter search grid for the *expr*$_{DGI}$ + *mut*$_{DGI, gene-level}$ + *cnv*$_{DGI}$ + *drugs*$_{ECFP4}$ model.

Hyperparameter	Values tested
expr_hlayers_sizes	[1024], [512], [256], [128], [64], [32], [16], [1024, 512], [512, 256], [256, 128], [128, 64], [64, 32], [32, 16], [1024, 512, 256], [512, 256, 128], [256, 128, 64], [128, 64, 32], [64, 32, 16]
drug_hlayers_sizes	[1024], [512], [256], [128], [64], [1024, 512], [512, 256], [256, 128], [128, 64], [1024, 512, 256], [512, 256, 128], [256, 128, 64]
mut_hlayers_sizes	[512], [256], [128], [64], [32], [16], [8], [512, 256], [256, 128], [128, 64], [64, 32], '[32, 16], [16, 8], [512, 256, 128], [256, 128, 64], [128, 64, 32], [64, 32, 16], [32, 16, 8]
cnv_hlayers_sizes	[1024], [512], [256], [128], [64], [32], [16], [1024, 512], [512, 256], [256, 128], [128, 64], [64, 32], [32, 16], [1024, 512, 256], [512, 256, 128], [256, 128, 64], [128, 64, 32], [64, 32, 16]
predictor_hlayers_sizes	[2048], [1024], [512], [256], [128], [64], [2048, 1024], [1024, 512], [512, 256], [256, 128], [128, 64], [2048, 1024, 512], [1024, 512, 256], [512, 256, 128], [256, 128, 64], [2048, 2048], [1024, 1024], [512, 512], [256, 256], [128, 128], [64, 64], [2048, 2048, 2048], [1024, 1024, 1024], [512, 512, 512], [256, 256, 256], [128, 128, 128], [64, 64, 64]
hidden_dropout	0.0, 0.1, 0.2, 0.3, 0.4, 0.5
hidden_activation	relu, leakyrelu, prelu
l2	0.00001, 0.0001, 0.001, 0.01, 0.1, 0
learn_rate	0.00001, 0.0001, 0.001, 0.01, 0.1

Table U – Hyperparameter search grid for the *expr*$_{DGI}$ + *mut*$_{pathway\text{-}level}$ + *cnv*$_{DGI}$ + *drugs*$_{ECFP4}$ model.

Hyperparameter	Values tested
expr_hlayers_sizes	[1024], [512], [256], [128], [64], [32], [16], [1024, 512], [512, 256], [256, 128], [128, 64], [64, 32], [32, 16], [1024, 512, 256], [512, 256, 128], [256, 128, 64], [128, 64, 32], [64, 32, 16]
drug_hlayers_sizes	[1024], [512], [256], [128], [64], [1024, 512], [512, 256], [256, 128], [128, 64], [1024, 512, 256], [512, 256, 128], [256, 128, 64]
mut_hlayers_sizes	[1024], [512], [256], [128], [64], [32], [16], [1024, 512], [512, 256], [256, 128], [128, 64], [64, 32], [32, 16], [1024, 512, 256], [512, 256, 128], [256, 128, 64], [128, 64, 32], [64, 32, 16]
cnv_hlayers_sizes	[1024], [512], [256], [128], [64], [32], [16], [1024, 512], [512, 256], [256, 128], [128, 64], [64, 32], [32, 16], [1024, 512, 256], [512, 256, 128], [256, 128, 64], [128, 64, 32], [64, 32, 16]
predictor_hlayers_sizes	[2048], [1024], [512], [256], [128], [64], [2048, 1024], [1024, 512], [512, 256], [256, 128], [128, 64], [2048, 1024, 512], [1024, 512, 256], [512, 256, 128], [256, 128, 64], [2048, 2048], [1024, 1024], [512, 512], [256, 256], [128, 128], [64, 64], [2048, 2048, 2048], [1024, 1024, 1024], [512, 512, 512], [256, 256, 256], [128, 128, 128], [64, 64, 64]
hidden_dropout	0.0, 0.1, 0.2, 0.3, 0.4, 0.5
hidden_activation	relu, leakyrelu, prelu
l2	0.00001, 0.0001, 0.001, 0.01, 0.1, 0
learn_rate	0.00001, 0.0001, 0.001, 0.01, 0.1

Table V – Hyperparameter search grid for the Elastic Net model.

Hyperparameter	Values tested
alpha	0.0001-1000
l1_ratio	0.1-0.9
max_iter	100000

Table W – Hyperparameter search grid for the LinearSVR model.

Hyperparameter	Values tested
C	0.0001-1000
epsilon	0.0001-10
loss	squared_epsilon_insensitive
dual	False
max_iter	100000

Table X – Hyperparameter search grid for the Nystroem+LinearSVR model.

Hyperparameter	Values tested
linearsvr__C	0.0001-1000
linearsvr__epsilon	0.0001-10
linearsvr__loss	squared_epsilon_insensitive
linearsvr__dual	False
linearsvr__max_iter	100000
nystroem__gamma	0.0001-1000
nystroem__n_components	25-150

Table Y – Hyperparameter search grid for the Random Forest model.

Hyperparameter	Values tested
n_estimators	100-1000
min_samples_split	2-5
max_depth	None, 5, 10, 15, 20
min_samples_leaf	1-5
max_features	auto, sqrt, log2

Table Z – Hyperparameter search grid for the XGBoost model.

Hyperparameter	Values tested
tree_method	hist
n_estimators	100-1000
learning_rate	0.0001-0.1
max_depth	3-9
min_child_weight	1-5
gamma	0-2
subsample	0.6-1.0

Table AA – Hyperparameter search grid for the LGBM model.

Hyperparameter	Values tested
n_estimators	100-1000
learning_rate	0.0001-0.1
max_depth	3-9
min_child_weight	1-5
min_split_gain	0-2
subsample	0.6-1.0
subsample_freq	0, 1, 5

Table AB – Hyperparameter values for the *cell line*$_{one\ hot}$ + *drugs*$_{one\ hot}$ model.

Hyperparameter	Values
expr_hlayers_sizes	[64, 32]
drug_hlayers_sizes	[64]
predictor_hlayers_sizes	[32, 32]
hidden_dropout	0.0
hidden_activation	leakyrelu
l2	0.0001
learn_rate	0.1
initializer	He normal
batchnorm	True
optimizer	Adam
loss	Mean squared error

Table AC – Hyperparameter values for the *cell line*$_{one\ hot}$ + *drugs*$_{ECFP4}$ model.

Hyperparameter	Values
expr_hlayers_sizes	[16]
drug_hlayers_sizes	[256, 128, 64]
predictor_hlayers_sizes	[256, 256]
hidden_dropout	0.0
hidden_activation	prelu
l2	0.0001
learn_rate	0.001
initializer	He normal
batchnorm	True
optimizer	Adam
loss	Mean squared error

Table AD – Hyperparameter values for the *expr*$_{DGI}$ + *drugs*$_{one\ hot}$ model.

Hyperparameter	Values
expr_hlayers_sizes	[1024, 512]
drug_hlayers_sizes	[16]
predictor_hlayers_sizes	[128, 64, 32]
hidden_dropout	0.0
hidden_activation	leakyrelu
l2	0.0001
learn_rate	0.01
initializer	He normal
batchnorm	True
optimizer	Adam
loss	Mean squared error

Table AE – Hyperparameter values for the *expr*$_{protein\ coding}$ + *drugs*$_{ECFP4}$ model.

Hyperparameter	Values
expr_hlayers_sizes	[256, 128]
drug_hlayers_sizes	[512, 256]
predictor_hlayers_sizes	[1024, 512, 256]
hidden_dropout	0.2
hidden_activation	leakyrelu
l2	0.0
learn_rate	0.0001
initializer	He normal
batchnorm	True
optimizer	Adam
loss	Mean squared error

Table AF – Hyperparameter values for the *expr*$_{protein\ coding,\ chromosome\ order\ 1D\ CNN}$ + *drugs*$_{ECFP4}$ model.

Hyperparameter	Values
expr_num_filters	[32, 32]
expr_kernel_sizes	[5, 5]
expr_pool_size	10
expr_batchnorm	True
drug_hlayers_sizes	[128]
predictor_hlayers_sizes	[256, 128, 64]
hidden_dropout (only fully connected subnetworks)	0.0
hidden_activation	leakyrelu
l2	0.001
learn_rate	0.0001
initializer	He normal
batchnorm (other subnetworks)	True
optimizer	Adam
loss	Mean squared error

Table AG – Hyperparameter values for the *expr*$_{\textit{protein coding, clustering order 1D CNN}}$ + *drugs*$_{\textit{ECFP4}}$ model.

Hyperparameter	Values
expr_num_filters	[64, 64]
expr_kernel_sizes	[5, 5]
expr_pool_size	5
expr_batchnorm	True
drug_hlayers_sizes	[256]
predictor_hlayers_sizes	[1024, 1024]
hidden_dropout (only fully connected subnetworks)	0.0
hidden_activation	leakyrelu
l2	0.0
learn_rate	0.001
initializer	He normal
batchnorm (other subnetworks)	True
optimizer	Adam
loss	Mean squared error

Table AH – Hyperparameter values for the *expr*$_{protein\ coding,\ chromosome\ order\ \ 2D\ CNN}$ + *drugs*$_{ECFP4}$ model.

Hyperparameter	Values
expr_num_filters	[32]
expr_kernel_size	(5, 5)
expr_pool_size	(2, 2)
expr_batchnorm	True
drug_hlayers_sizes	[128, 64]
predictor_hlayers_sizes	[4096]
hidden_dropout (only fully connected subnetworks)	0.0
hidden_activation	prelu
l2	0.01
learn_rate	0.001
initializer	He normal
batchnorm (other subnetworks)	True
optimizer	Adam
loss	Mean squared error

Table AI – Hyperparameter values for the *expr*$_{\textit{protein coding, clustering order 2D CNN}}$ + *drugs*$_{\textit{ECFP4}}$ model.

Hyperparameter	Values
expr_num_filters	[16, 32, 64]
expr_kernel_size	(3, 3)
expr_pool_size	(2, 2)
expr_batchnorm	False
drug_hlayers_sizes	[512]
predictor_hlayers_sizes	[256, 128, 64]
hidden_dropout (only fully connected subnetworks)	0.1
hidden_activation	relu
l2	0.1
learn_rate	0.0001
initializer	He normal
batchnorm (other subnetworks)	True
optimizer	Adam
loss	Mean squared error

Table AJ – Hyperparameter values for the *expr*$_{landmark}$ + *drugs*$_{ECFP4}$ model.

Hyperparameter	Values
expr_hlayers_sizes	[512, 256, 128]
drug_hlayers_sizes	[1024, 512]
predictor_hlayers_sizes	[1024, 1024, 1024]
hidden_dropout	0.1
hidden_activation	relu
l2	0.00001
learn_rate	0.0001
initializer	He normal
batchnorm	True
optimizer	Adam
loss	Mean squared error

Table AK – Hyperparameter values for the $\textit{expr}_{DGI}$ + $\textit{drugs}_{ECFP4}$ model.

Hyperparameter	Values
expr_hlayers_sizes	[1024, 512, 256]
drug_hlayers_sizes	[512]
predictor_hlayers_sizes	[512, 256]
hidden_dropout	0.1
hidden_activation	prelu
l2	0.1
learn_rate	0.0001
initializer	He normal
batchnorm	True
optimizer	Adam
loss	Mean squared error

Table AL – Hyperparameter values for the *$expr_{COSMIC}$* + *$drugs_{ECFP4}$* model.

Hyperparameter	Values
expr_hlayers_sizes	[64]
drug_hlayers_sizes	[1024, 512, 256]
predictor_hlayers_sizes	[2048]
hidden_dropout	0.1
hidden_activation	prelu
l2	0.001
learn_rate	0.0001
initializer	He normal
batchnorm	True
optimizer	Adam
loss	Mean squared error

Table AM – Hyperparameter values for the *expr*$_{NCG}$ + *drugs*$_{ECFP4}$ model.

Hyperparameter	Values
expr_hlayers_sizes	[1024]
drug_hlayers_sizes	[256]
predictor_hlayers_sizes	[256, 256, 256]
hidden_dropout	0.0
hidden_activation	leakyrelu
l2	0.001
learn_rate	0.0001
initializer	He normal
batchnorm	True
optimizer	Adam
loss	Mean squared error

Table AN – Hyperparameter values for the *expr*$_{DGI + landmark}$ + *drugs*$_{ECFP4}$ model.

Hyperparameter	Values
expr_hlayers_sizes	[64, 32]
drug_hlayers_sizes	[1024]
predictor_hlayers_sizes	[256, 256]
hidden_dropout	0.1
hidden_activation	prelu
l2	0.0
learn_rate	0.01
initializer	He normal
batchnorm	True
optimizer	Adam
loss	Mean squared error

Table AO – Hyperparameter values for the *$expr_{DGI + NCG}$* + *$drugs_{ECFP4}$* model.

Hyperparameter	Values
expr_hlayers_sizes	[256]
drug_hlayers_sizes	[512]
predictor_hlayers_sizes	[512]
hidden_dropout	0.4
hidden_activation	prelu
l2	0.0001
learn_rate	0.001
initializer	He normal
batchnorm	True
optimizer	Adam
loss	Mean squared error

Table AP – Hyperparameter values for the $expr_{UMAP}$ + $drugs_{ECFP4}$ model.

Hyperparameter	Values
expr_hlayers_sizes	[32, 16]
drug_hlayers_sizes	[64]
predictor_hlayers_sizes	[1024, 512]
hidden_dropout	0.0
hidden_activation	prelu
l2	0.001
learn_rate	0.001
initializer	He normal
batchnorm	True
optimizer	Adam
loss	Mean squared error

Table AQ – Hyperparameter values for the *expr*$_{WGCNA}$ + *drugs*$_{ECFP4}$ model.

Hyperparameter	Values
expr_hlayers_sizes	[64, 32]
drug_hlayers_sizes	[256]
predictor_hlayers_sizes	[1024, 512]
hidden_dropout	0.5
hidden_activation	prelu
l2	0.0001
learn_rate	0.001
initializer	He normal
batchnorm	True
optimizer	Adam
loss	Mean squared error

Table AR – Hyperparameter values for the *expr*$_{DGI}$ + *drugs*$_{LayeredFP}$ model.

Hyperparameter	Values
expr_hlayers_sizes	[64]
drug_hlayers_sizes	[1024]
predictor_hlayers_sizes	[2048, 1024, 512]
hidden_dropout	0.0
hidden_activation	leakyrelu
l2	0.0001
learn_rate	0.001
initializer	He normal
batchnorm	True
optimizer	Adam
loss	Mean squared error

Table AS – Hyperparameter values for the *expr*$_{DGI}$ + *drugs*$_{TextCNN}$ model.

Hyperparameter	Values
expr_hlayers_sizes	[256, 128]
drug_dropout	0.5
drug_kernel_sizes	[3, 5, 7]
drug_n_embedding	75
drug_num_filters	[32, 32, 32]
predictor_hlayers_sizes	[512]
hidden_dropout (other subnetworks)	0.2
hidden_activation (except for drug subnetworks)	prelu
TextCNN activation	None (Conv1D layers), ‘relu’ (Dense layer)
l2	0.1
learn_rate	0.001
initializer	He normal
batchnorm (only fully connected subnetworks	True
optimizer	Adam
loss	Mean squared error

Table AT – Hyperparameter values for the *$expr_{DGI}$* + *$drugs_{GCN}$* model.

**Hyperparameter**	**Values**
expr_hlayers_sizes	[512, 256, 128]
drug_gcn_layers	[128, 128]
drug_residual_connection	True
drug_dropout	0.0
predictor_hlayers_sizes	[2048, 2048]
hidden_dropout (other subnetworks)	0.3
hidden_activation (except for drug subnetworks)	prelu
GCN activation	relu
l2	0.001
learn_rate	0.001
initializer	He normal
batchnorm (only fully connected subnetworks)	True
optimizer	Adam
loss	Mean squared error

Table AU – Hyperparameter values for the *expr*$_{DGI}$ + *drugs*$_{GAT}$ model.

Hyperparameter	Values
expr_hlayers_sizes	[64, 32, 16]
drug_gat_layers	[8, 8, 8]
drug_num_attention_heads	6
drug_concat_heads	True
drug_residual_connection	False
drug_dropout	0.0
predictor_hlayers_sizes	[1024, 1024, 1024]
hidden_dropout (other subnetworks)	0.0
hidden_activation (except for drug subnetworks)	prelu
GAT activation	elu
l2	0.0001
learn_rate	0.0001
initializer	He normal
batchnorm (only fully connected subnetworks)	True
optimizer	Adam
loss	Mean squared error

Table AV – Hyperparameter values for the *expr*$_{DGI}$ + *drugs*$_{MTE}$ model.

Hyperparameter	Values
expr_hlayers_sizes	[16]
drug_hlayers_sizes	[256]
predictor_hlayers_sizes	[512, 512, 512]
hidden_dropout	0.0
hidden_activation	leakyrelu
l2	0.0
learn_rate	0.0001
initializer	He normal
batchnorm	True
optimizer	Adam
loss	Mean squared error

Table AW – Hyperparameter values for the $expr_{DGI}$ + $mut_{DGI,\ gene\text{-}level}$ + cnv_{DGI} + $drugs_{ECFP4}$ model.

Hyperparameter	Values
expr_hlayers_sizes	[128, 64]
drug_hlayers_sizes	[512]
mut_hlayers_sizes	[256]
cnv_hlayers_sizes	[32, 16]
predictor_hlayers_sizes	[256]
hidden_dropout	0.0
hidden_activation	prelu
l2	0.0001
learn_rate	0.001
initializer	He normal
batchnorm	True
optimizer	Adam
loss	Mean squared error

Table AX – Hyperparameter values for the *expr*$_{DGI}$ + *mut*$_{pathway\text{-}level}$ + *cnv*$_{DGI}$ + *drugs*$_{ECFP4}$ model.

Hyperparameter	Values
expr_hlayers_sizes	[64]
drug_hlayers_sizes	[512]
mut_hlayers_sizes	[256]
cnv_hlayers_sizes	[256, 128, 64]
predictor_hlayers_sizes	[1024]
hidden_dropout	0.1
hidden_activation	prelu
l2	0.0
learn_rate	0.0001
initializer	He normal
batchnorm	True
optimizer	Adam
loss	Mean squared error

Table AY – Hyperparameter values for the Elastic Net model.

Hyperparameter	Values
alpha	0.00029321308659580964
l1_ratio	0.8905437519154037
max_iter	100000

Table AZ – Hyperparameter values for the LinearSVR model.

Hyperparameter	Values
C	7.8705199326554025
epsilon	0.10342495585470976
loss	squared_epsilon_insensitive
dual	False
max_iter	100000

Table BA – Hyperparameter values for the Nystroem+LinearSVR model.

Hyperparameter	Values
linearsvr__C	13.558053311806814
linearsvr__epsilon	0.019433401328014608
linearsvr__loss	squared_epsilon_insensitive
linearsvr__dual	False
linearsvr__max_iter	100000
nystroem__gamma	0.0007899335083362674
nystroem__n_components	150

Table BB – Hyperparameter values for the Random Forest model.

Hyperparameter	Values
n_estimators	863
min_samples_split	5.0
max_depth	None
min_samples_leaf	2.0
max_features	sqrt

Table BC – Hyperparameter values for the XGBoost model.

Hyperparameter	Values
tree_method	hist
n_estimators	615
learning_rate	0.015408845208944859
max_depth	8
min_child_weight	5
gamma	1.0762624813641142
subsample	0.7014936347327818

Table BD – Hyperparameter values for the LGBM model.

Hyperparameter	Values
n_estimators	972
learning_rate	0.05107911749137478
max_depth	8
min_child_weight	3
min_split_gain	0.6878242640017198
subsample	0.6730297748696231
subsample_freq	5.0
